# The Underestimated Role of Iron in Frontotemporal Dementia: A Narrative Review

**DOI:** 10.3390/ijms252312987

**Published:** 2024-12-03

**Authors:** Sara Ferretti, Isabella Zanella

**Affiliations:** 1 Department of Molecular and Translational Medicine, University of Brescia, 25123 Brescia, Italy; s.ferretti002@studenti.unibs.it; 2 Medical Genetics Laboratory, Diagnostic Department, ASST Spedali Civili di Brescia, 25123 Brescia, Italy

**Keywords:** frontotemporal dementia (FTD), primary progressive aphasia (PPA), microtubule-associated protein tau (MAPT/TAU), progranulin (GRN), chromosome 9 open reading frame 72 (C9orf72), iron homeostasis, reactive oxygen species (ROS), mitochondrial dysfunction, protein aggregation, ferroptosis

## Abstract

The term frontotemporal dementia (FTD) comprises a group of neurodegenerative disorders characterized by the progressive degeneration of the frontal and temporal lobes of the brain with language impairment and changes in cognitive, behavioral and executive functions, and in some cases motor manifestations. A high proportion of FTD cases are due to genetic mutations and inherited in an autosomal-dominant manner with variable penetrance depending on the implicated gene. Iron is a crucial microelement that is involved in several cellular essential functions in the whole body and plays additional specialized roles in the central nervous system (CNS) mainly through its redox-cycling properties. Such a feature may be harmful under aerobic conditions, since it may lead to the generation of highly reactive hydroxyl radicals. Dysfunctions of iron homeostasis in the CNS are indeed involved in several neurodegenerative disorders, although it is still challenging to determine whether the dyshomeostasis of this essential but harmful metal is a direct cause of neurodegeneration, a contributor factor or simply a consequence of other neurodegenerative mechanisms. Unlike many other neurodegenerative disorders, evidence of the dysfunction in brain iron homeostasis in FTD is still scarce; nonetheless, the recent literature intriguingly suggests its possible involvement. The present review aims to summarize what is currently known about the contribution of iron dyshomeostasis in FTD based on clinical, imaging, histological, biochemical and molecular studies, further suggesting new perspectives and offering new insights for future investigations on this underexplored field of research.

## 1. Introduction

Frontotemporal lobar degeneration (FTLD) is the most common cause of early onset dementia after Alzheimer’s disease (AD) and Dementia with Lewy Bodies (DLB). FTLD defines a large and heterogeneous group of neurodegenerative disorders, characterized by the progressive degeneration of the frontal and temporal lobes of the brain, leading to the atrophy of these regions. The FTLD spectrum encompasses several disorders: frontotemporal dementia (FTD), including the FTD behavioral variant (bv-FTD), the most common form of FTLD, and the semantic and non-fluent variants of primary progressive aphasia (sv-PPA and nfv-PPA); FTD with amyotrophic lateral sclerosis/motor neuron disease (FTD-ALS/MND); and the extrapyramidal atypical parkinsonian syndromes progressive supranuclear palsy (PSP) and corticobasal syndrome (CBS) [1].

From a pathological point of view, FTLDs are proteinopathies characterized by the presence of intracellular aggregates of proteins within neurons of the affected regions. Different proteins are involved and, on the basis of the type of proteinaceous aggregates, FTLDs can be further distinguished in FTLD with aggregates of the microtubule-associated protein tau (*MAPT/TAU*) (FTLD-tau); FTLD with TAR DNA binding protein (*TARDBP/TDP43*) inclusions (FTLD-TDP); and FTLD with fused in sarcoma (*FUS*) aggregates (FTLD-FUS) or, more generally speaking, FTLD with aggregation of members of the FET protein family, including *FUS*, EWS RNA binding protein 1 (*EWSR1*) and TATA-box binding protein associated factor 15 (*TAF15*) (FTLD-FET) [2].

About 30–50% of FTLD cases have a positive family history of dementia or other neurodegenerative diseases (familial FTLD, f-FTLD). In a high proportion, FTLD is inherited in an autosomal dominant manner [2]. Mutations in three genes account for the majority of known f-FTLDs: MAPT/TAU, which is mainly associated with tau pathology, and progranulin (GRN) and chromosome 9 open reading frame 72 (C9orf72) genes, which are both characterized by the presence of *TARDBP/TDP43* inclusions.

Although the main symptoms of FTD are cognitive, behavioral or linked to speech impairment, FTD can also manifest with motor dysfunctions, which are characterized by the degeneration of upper and lower motor neurons like in ALS/MND or, conversely, patients with ALS/MND frequently show some degree of cognitive and behavioral impairment, fitting with FTD. It is currently recognized that ALS/MND and FTD represent a disease spectrum based on several clinical, pathological and genetic features, considering the overlapping symptoms, the shared pathology with the presence of the same proteinaceous aggregates and the evidence of common associated genetic variants like the hexanucleotide large expansion in the C9orf72 gene [3].

**Figure 1:**
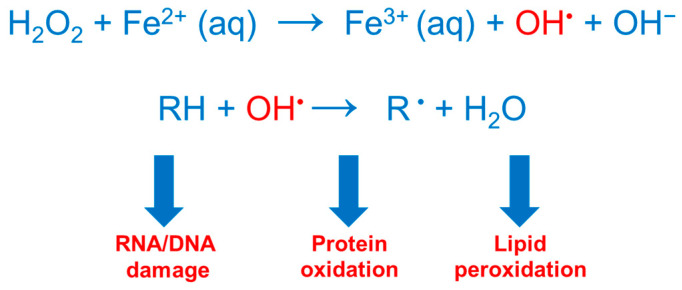
The Fenton reaction is a chemical reaction in which an important role is played by Fe^2+^. Once entered in the cells, redox-active Fe^2+^ is trafficked to the sites of utilization or storage as a cytosolic labile iron pool (LIP), probably vehicled by low molecular weight molecules like citrate or by chaperone proteins (see below in the text) to avoid harmful reactions. Fe^2+^ and hydrogen peroxide can indeed catalyze the generation of strong oxidizing species, the hydroxyl radicals OH^•^, which are capable of oxidizing a wide variety of organic compounds and biomolecules, like lipids, proteins and nucleic acids, ultimately causing cell death and tissue damage [4].

Iron is a crucial microelement that acts as a co-factor of many enzymes and proteins involved in a variety of different essential cellular functions from DNA synthesis and repair to cell proliferation and differentiation, oxygen transport, mitochondrial respiration and, characteristically in the nervous system, neurotransmitter synthesis and myelination. In most of these functions, the interconversion ability of this transition metal between the reduced ferrous iron (Fe^2+^) and the oxidized ferric (Fe^3+^) state is central. However, if not tightly controlled, iron redox cycling may be very harmful under aerobic conditions, since it can generate highly reactive hydroxyl radicals through the Fenton reaction, resulting in the oxidative damage of lipids, proteins and nucleic acids, and ultimately causing cell death through several pathways and tissue damage [4] (Figure 1).

For these reasons, organisms and cells have evolved a complex network of specific proteins that safely handle and control iron in the body and all cells to maintain its homeostasis and avoid its deficiency, excess or maldistribution. If correct iron management and balancing is necessary in the whole organism, it is also particularly important in the central nervous system (CNS), which needs iron for several fundamental functions. Perinatal iron deficiency indeed results in compromised brain energy metabolism, the synthesis of neurotransmitters, myelination, neuronal growth and differentiation, and it is associated with neurodevelopmental impairment and long-term effects on cognition [5]. Iron deficiency, excess or dyshomeostasis have also been described in several neurodegenerative disorders, like AD, Parkinson’s disease (PD), neurodegenerations with brain iron accumulation (NBIAs) and ALS/MND [6].

However, little is known regarding the role of iron dyshomeostasis in FTD, although in more recent years, some intriguing data have begun to appear in the literature. This review aims to provide an overview of currently published data to date on this topic, also aiming to offer new insights for future investigations.

## 2. The Biochemistry of Iron

**Figure 2:**
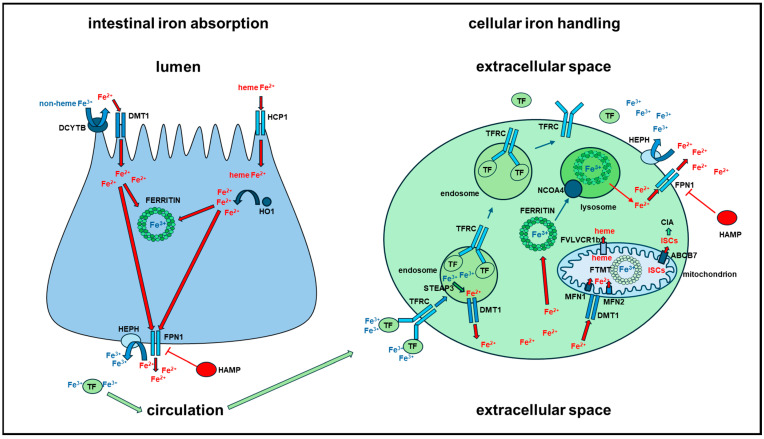
Intestinal iron absorption (**left**) and iron handling in a generic cell (**right**). Duodenal enterocytes acquire dietary iron both as inorganic iron and heme iron. At the luminal membrane, inorganic ferric iron (Fe^3+^) is reduced to ferrous iron (Fe^2+^) by the duodenal cytochrome b/cytochrome b reductase (*DCYTB/CYBRD1*) and transported into the cell by divalent metal transporter 1/solute carrier family 11 member 2 (*DMT1/SLC11A2*). Heme iron is internalized through the heme carrier protein 1/solute carrier family 46 member 1 (*HCP1/SLC46A1*) and released in the cytoplasm as Fe^2+^ by heme oxygenase 1 (*HO1/HMOX1*). Enterocytes store iron within ferritin or export iron at the basolateral membrane through ferroportin1/solute carrier family 40 member 1 (*FPN1/SLC40A1*), whose function is negatively regulated by the master regulator of iron homeostasis hepcidin (*HAMP*), which is mainly secreted by hepatocytes. Iron released in the extracellular space is then oxidized by hephaestin (*HEPH*) and loaded onto transferrin (*TF*), which distributes iron as Fe^3+^ to tissues and cells through the circulation. In most cells, holo-*TF* interacts with the ubiquitous cell surface transferrin receptor (*TFRC*) and the complex is internalized by endocytosis. Within the endosomes, iron is released by acidification, reduced to Fe^2+^ by the six-transmembrane epithelial antigen of prostate 3 (*STEAP3*) ferrireductase and released in the cytoplasm by the divalent metal transporter 1/solute carrier family 11 member 2 (*DMT1/SLC11A2*). The apo-*TF*/*TFRC* complex is recycled to the cell surface and dissociated; *TFRC* remains in the membrane for further cycles of iron uptake, while *TF* is recycled in the circulation. Cytosolic iron is then delivered to the sites of usage or stored within ferritin. Mitochondria are the main site of iron cellular usage. Iron enters mitochondria through *DMT1/SLC11A2*, mitoferrin 1/solute carrier family 25 member 37 (*MFN1/SLC25A37*) and mitoferrin 2/solute carrier family 25 member 28 (*MFN2/SLC25A28*), and it is mainly used for the synthesis of heme and iron sulfur clusters (ISCs). Newly synthesized heme is transferred to mitochondrial heme-containing proteins or transported to the cytosol for transfer to cytosolic heme-containing proteins through the mitochondrial heme transporter feline leukemia virus subgroup C receptor 1b (*FLVCR1b).* ISCs are assembled partly in the mitochondrion by the mitochondrial ISC assembly machinery and partly in the cytosol by the cytosolic ISC assembly machinery (CIA), which receives ISCs from mitochondria through the ATP binding cassette subfamily B member 7 (*ABCB7*) transporter. Excess mitochondrial iron is stored in mitochondrial ferritin (*FTMT*), while excess cytosolic iron is mainly stored in cytosolic ferritin, that may quickly release it for cellular needs through nuclear receptor coactivator 4 (*NCOA4*)-mediated ferritinophagy or by an autophagy-independent lysosomal pathway. Cellular iron excess may also exit cells through *FPN1/SLC40A1*, which is coupled with ferroxidase proteins like ceruloplasmin (*CP*) and *HEPH*.

**Figure 3:**
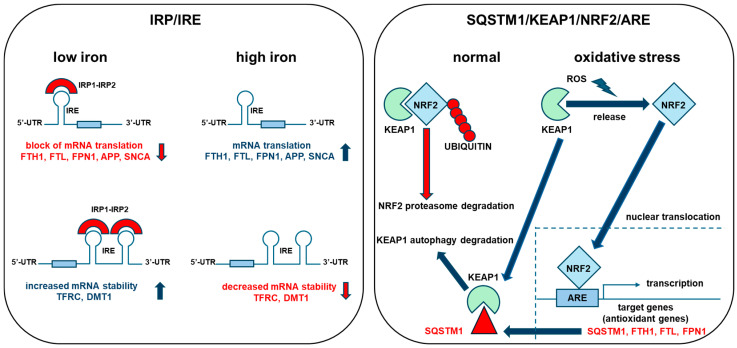
Regulation of iron homeostasis by cellular iron levels (**left**) and oxidative stress (**right**). Iron regulatory protein 1/aconitase 1 (*IRP1/ACO1*) and iron regulatory protein 2/iron responsive element-binding protein 2 (*IRP2/IREB2*) ubiquitously regulate the transcription of several iron-related genes. In conditions of low cellular iron levels, both proteins recognize and bind RNA structures named iron-responsive elements (IREs) within the 5′- or 3′-untranslated regions (UTRs) of the regulated mRNAs. In this way, the translation of ferritin heavy chain 1 (FTH1), ferritin light chain (FTL) and ferroportin1/solute carrier family 40 member 1 (FPN1/SLC40A1) mRNAs is blocked, while transferrin receptor (TFRC) and divalent metal transporter 1/solute carrier family 11 member 2 (DMT1/SLC11A2) mRNAs are stabilized and translated, thus decreasing cellular iron storage and release and increasing iron uptake. On the contrary, in iron excess conditions, both IRPs release IREs, increasing the translation of FTH1, FTL and FPN1/SLC40A1 mRNAs while decreasing the stability of TFRC and DMT1/SLC11A2 mRNAs; in this way, cellular iron storage and release are increased, while uptake is decreased. Amyloid beta precursor protein (APP) and synuclein alpha (SNCA) genes are also involved in iron homeostasis, the first being a ferroxidase and the second a ferrireductase, and they are both regulated by IRPs, like FTH1, FTL and FPN1/SLC40A1 mRNAs. Some iron-related genes are also regulated by oxidative stress through the sequestosome 1/protein 62/kelch-like ECH associated protein 1/NFE2 like bZIP transcription factor 2 (*SQSTM1/p62/KEAP1/NRF2*) signaling pathway. Under normal conditions, *KEAP1* interacts with and directs ubiquitinated *NRF2* to proteasomal degradation; under oxidative stress conditions, *KEAP1* releases *NRF2*, the transcription factor translocates to the nucleus and recognizes specific DNA sequences, the antioxidant responsive elements (AREs), in the promoter region of several genes involved in the antioxidant response, among which include FTH1, FTL, FPN1/SLC40A1 and SQSTM1/p62. *SQSTM1/p62* also binds and directs *KEAP1* to autophagic degradation, further activating *NRF2*-related antioxidant response.

**Figure 4:**
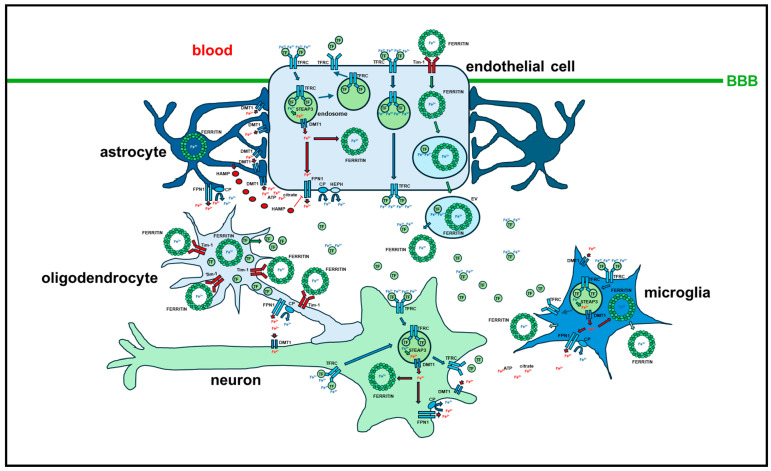
Iron handling in the brain. Iron entry into the brain is strictly regulated at the blood–brain barrier (BBB) by the polarized endothelial cells (ECs), which is supported by astrocytes. Iron enters ECs through the classical transferrin/transferrin receptor (*TF/TFRC*) endocytic pathway and is released by divalent metal transporter 1/solute carrier family 11 member 2 (*DMT1/SLC11A2*) in the cytosol after its reduction by six-transmembrane epithelial antigen of prostate 3 (*STEAP3*) within endosomes. The apo-*TF*/*TFRC* complex is then recycled to the cell surface and dissociated; *TFRC* remains in the membrane for further cycles of iron uptake, while *TF* is recycled in the circulation. Alternative routes for iron entry in ECs are the transcytosis of holo-*TF* from the luminal to the abluminal side of ECs or the uptake of ferritin by *TFRC* or T-cell immunoglobulin mucin domain 1 protein (Tim-1). Ferritin and *TF* may be released at the abluminal side of ECs through extracellular vesicles (EVs). In ECs, iron may be stored within the ferritin cage or released at the abluminal side through ferroportin1/solute carrier family 40 member 1 (*FPN1/SLC40A1*), which is coupled with ferroxidase proteins like ceruloplasmin (*CP*) and hephaestin (*HEPH*). Astrocytes and choroid plexus express and release hepcidin (*HAMP*), in this way controlling iron entry in the brain through its interaction with *FPN1/SLC40A1.* The control of iron entry is also performed by apo- and holo-*TF* levels in the extracellular space through the regulation of *FPN1/SLC40A1* stability and *HEPH* activity. Astrocytes, in direct contact with ECs, uptake iron through *DMT1/SLC11A2* and then redistribute the metal in the extracellular space through *FPN1/SLC40A1* coupled with *CP.* Iron moves in the brain extracellular space bound to citrate or ATP (released by astrocytes) or to *TF* (mainly secreted by oligodendrocytes and the choroid plexus). Mature oligodendrocytes acquire iron through ferritin uptake by Tim-1, store iron in ferritin and release iron through *FPN1/SLC40A1*. Microglial cells acquire iron through *TF/TFR*C endocytosis and through *DMT1/SLC11A2*, store the metal in the ferritin shell and release iron through *FPN1/SLC40A1* and secreting ferritin. Like for microglia, iron uptake is obtained through *TF/TFR*C endocytosis and through *DMT1/SLC11A2* in neurons, which store small amounts of iron in ferritin and release excess iron through *FPN1/SLC40A1*.

As already mentioned, thanks to redox cycling between the reduced Fe^2+^ and the oxidized Fe^3+^ states, iron plays a significant role in several cellular functions in the whole body and particularly in the brain, where it is also peculiarly engaged as a co-factor of several enzymes and proteins involved in myelination, synapse development and the synthesis of several neurotransmitters, like dopamine and norepinephrine. Due to its potentially harmful redox cycling, iron levels in all tissues and organs are finely regulated though several strategies. Here, we review iron homeostasis in the whole body (Figure 2 and Figure 3), which is comprehensively described in more detail in several excellent publications [7,8,9], and then we focus on the brain (Figure 4).

Body iron is mainly recycled through the phagocytosis of aged erythrocytes and free iron release from hemoglobin by macrophages, its re-use for erythropoiesis or its reversible storage in the liver; its little daily loss (1–2 mg/day) is counterbalanced by intestine absorption through the diet. Dietary iron is absorbed by duodenal enterocytes in a strictly controlled way to avoid its body accumulation. To make up for the iron daily loss, enterocyte can acquire dietary iron as heme iron or inorganic iron through distinct pathways, namely the heme carrier protein 1/solute carrier family 46 member 1 (*HCP1/SLC46A1*) coupled to heme oxygenase 1 (*HO1/HMOX1*) or the duodenal cytochrome b/cytochrome b reductase (*DCYTB/CYBRD1*) coupled to divalent metal transporter 1/solute carrier family 11 member 2 (*DMT1/SLC11A2*), respectively. Once in the enterocytes, iron is stored within the main iron storage protein ferritin or exported to the plasma for its utilization in the whole body. Iron exits the enterocytes through ferroportin1/solute carrier family 40 member 1 (*FPN1/SLC40A1*) at the enterocyte basolateral membrane, it is oxidized by hephaestin (*HEPH*) and loaded onto transferrin (*TF*), which carries and safely distributes iron as Fe^3+^ to other organs, tissues and cells through circulation. Iron exit from enterocytes is strictly controlled by the iron regulatory hormone hepcidin (*HAMP*), which is mainly secreted by hepatocytes. Circulating *HAMP* can bind *FPN1/SLC40A1*, leading to its degradation and negatively regulating iron export from enterocytes (Figure 2).

Iron, vehicled through the circulation, is mainly acquired by cells through the interaction of iron-loaded-*TF* (holo-*TF*) and the ubiquitous cell surface transferrin receptor (*TFRC*). The iron-loaded *TF*/*TFRC* complex is internalized by endocytosis, conveyed to the endosomes and released within endosomes by acidification. The discharged *TF*/*TFRC* complex is recycled to the cell surface; *TF* is dissociated from *TFRC* and recycled into the bloodstream as apo-*TF*. Within the endosome, Fe^3+^ is reduced to Fe^2+^ by the six-transmembrane epithelial antigen of prostate 3 (*STEAP3*) ferrireductase and then released into the cytosol by *DMT1*/*SLC11A2*. Cytosolic iron is delivered to the main cellular iron usage sites, like mitochondria, where it is used for the synthesis of iron–sulfur clusters (ISCs) and heme, or it is stored within the main iron storage protein ferritin for later use. Several alternative mechanisms of cellular iron uptake are known, like extracellular ferritin intake by the scavenger receptor class A member 5 (*SCARA5*) receptor or by *TFRC* itself or the non-transferrin bound iron intake through *DCYTB/CYBRD1* coupled to *DMT1/SLC11A2*. The trafficking of harmful Fe^2+^ through the cytosol to ferritin or to the sites of its utilization is only partially known. Cytosolic labile iron pool (LIP) is most probably vehicled by low molecular weight molecules like citrate or by chaperone proteins (Figure 2).

To enter mitochondria, iron must cross the outer and inner mitochondrial membranes (OMM and IMM). *DMT1/SLC11A2* seems to be involved in the uptake at the OMM, while iron traverses the IMM through members of the SLC family, such as mitoferrin 1/solute carrier family 25 member 37 (*MFN1/SLC25A37*) and mitoferrin 2/solute carrier family 25 member 28 (*MFN2/SLC25A28*). Once in the mitochondrial matrix, iron is mainly used for the synthesis of ISCs and heme. Several proteins are engaged in the ISC assembly machinery. ISCs are assembled on scaffold proteins and then incorporated into apoproteins, which are involved in several essential cellular processes. In doing this, the mitochondrial machinery involved in ISC synthesis is supported also by the cytosolic ISC assembly machinery (CIA), which receives ISCs from mitochondria through the ATP binding cassette subfamily B member 7 (*ABCB7*) transporter. The heme synthesis pathway begins in the mitochondrion, continues in the cytosol and terminates again in the mitochondrion with the insertion of Fe^2+^ into protoporphyrin IX by ferrochelatase (*FECH*). Heme is transferred to mitochondrial heme-containing proteins or transported to the cytosol for transfer to cytosolic heme-containing proteins through the mitochondrial heme transporter feline leukemia virus subgroup C receptor 1b (*FLVCR1b)* (Figure 2).

As already mentioned, the excess cytosolic iron is deposited as non-reactive but bio-available iron oxide within ferritin, which is the ubiquitous cellular iron storage protein. Ferritin is a 24-subunit heteropolymer composed of ferritin heavy chain 1 (*FTH1*) and ferritin light chain (*FTL*) proteins that co-assemble to make a spherical shell in reciprocal ratios that vary among different tissues. The heteropolymer can bind up to 4500 iron atoms within its cavity. *FTH1* has ferroxidase activity, crucially involved in iron conversion in its oxidized Fe^3+^ form, necessary for its deposition in the ferritin cavity, while *FTL* has a nucleation site in which Fe^3+^ binds, promoting the creation of the ferritin core (Figure 2).

When in excess, mitochondrial iron is conversely stored in a specific ferritin named mitochondrial ferritin (*FTMT*). Within the mitochondria, *FTMT* assembles as a 24-subunit homopolymer molecule that has ferroxidase activity and incorporates iron similarly to the cytosolic heteropolymer. *FTMT* expression is, however, restricted in specific cell types and tissues, which are all characterized by high oxidative metabolic activity, like testis, heart, kidney and some areas of the brain, but it has not been observed in iron storage tissues like the liver and the spleen. The main physiological role of this specific ferritin is the regulation of mitochondrial iron availability and the control of oxidative damage (Figure 2).

Under basal or iron depletion conditions, iron deposited in the cytosolic ferritin shell may be quickly mobilized for cellular needs by the nuclear receptor coactivator 4 (*NCOA4*)-mediated ferritinophagy or by an autophagy-independent lysosomal pathway. Excess cellular iron may also be exported by the iron exporter ferroportin1/solute carrier family 40 member 1 (*FPN1/SLC40A1*) coupled with ferroxidase proteins like ceruloplasmin (*CP*) and hephaestin (*HEPH*). Iron exit from cells is regulated both in a systemic way and, in some cells like cardiomyocytes, adipocytes, pancreatic β-cells and astrocytes, in an autocrine/paracrine way by the iron regulatory hormone *HAMP*, which is mainly derived from the liver but is also produced and released by further tissues and cells like those mentioned [7] (Figure 2).

As highlighted above, cellular iron homeostasis is finely regulated in all body cells to avoid iron load or deficiency and to prevent its harmful redox cycling. This tight control is ubiquitously and finely regulated by the iron regulatory proteins (*IRPs*), namely the iron regulatory protein 1/aconitase 1 (*IRP1/ACO1*) and the iron regulatory protein 2/iron responsive element-binding protein 2 (*IRP2/IREB2*), which are both regulated by cellular iron levels in their functions. Both proteins bind to specific RNA structures named iron-responsive elements (IREs), located within the 5′- or 3′-untranslated regions (UTRs) of several mRNAs codifying for iron-related proteins, finely regulating their expression based on cellular iron needs. In iron starvation conditions, the binding of *IRPs* to the 5′-UTR located IREs blocks the translation of genes like FTH1, FTL and FPN1/SLC40A1, repressing iron storage and cellular iron release, while binding to the 3′-UTR located IREs stabilizes mRNAs like those specifying TFRC and DMT1/SLC11A2 genes and facilitates their translation, inducing cellular iron uptake. On the contrary, in high iron conditions, *IRPs* do not bind IREs (*IRP1/ACO1* functions as cytosolic aconitase and *IRP2/IREB2* is degraded by the proteasome) and act to enhance cytosolic ferritin expression, iron accumulation in its shells and cellular iron export through *FPN1/SLC40A1* while repressing cellular iron uptake through *TFRC* and *DMT1/SLC11A2* [9]. Furthermore, several genes that are crucial in maintaining iron homeostasis and in avoiding iron-induced cellular damage are also regulated by the kelch-like ECH associated protein 1/NFE2 like bZIP transcription factor 2 (*KEAP1/NRF2*) signaling pathway. *NRF2* is a ubiquitously expressed transcription factor involved in sensing oxygen radicals generated by iron redox activity. Under normal conditions, *NRF2* is degraded by proteasomal degradation through the interaction with the E3 ubiquitin ligase adaptor *KEAP1* while, under oxidative stress conditions, *NRF2* ubiquitination and degradation by *KEAP1* is inhibited, causing *NRF2* accumulation and its translocation to the nucleus, where the transcription factor promotes the expression of genes involved in the cellular antioxidant and detoxification response [10]. These genes harbor an antioxidant responsive element (ARE) in the promoter region that is recognized by *NRF2* and upregulates their transcription [11]. These genes include FTH1, FTL and FPN1/SLC40A1 [7,12] (Figure 3).

Although the described general mechanisms of cellular iron uptake, cytosolic handling and export are also found in the CNS, iron homeostasis in the brain is largely independent of its systemic regulation. First, iron import in the CNS is regulated by the polarized endothelial cells (ECs) of the blood–brain barrier (BBB), which is supported by astrocytes. Holo-*TF* is endocytosed by *TFRC* on the luminal side of ECs and handled in the endosomes as described above by *STEAP3* and *DMT1/SLC11A2*. Once released in the cytosol as Fe^2+^, iron is stored in ferritin or released by *FPN1/SLC40A1* through the abluminal membrane, where ferroxidases like *CP* or *HEPH* convert Fe^2+^ to Fe^3+^. Astrocytes are intimately in contact with ECs through their endfeet and mainly acquire released iron through *DMT1/SLC11A2* on their plasma membrane, redistributing iron in the extracellular space through *FPN1/SLC40A1* coupled with *CP*. Released iron binds low molecular weight molecules like citrate or extracellular *TF* and is then uptaken by neurons and glial cells for their needs. *TF* in the extracellular space is mainly produced and secreted by oligodendrocytes and the choroid plexus, and it is only minimally derived from blood. Alternative routes to the endocytosis of the holo-*TF*/*TFRC* complex for brain iron uptake by ECs are the transcytosis of holo-*TF* from the luminal to the abluminal side of ECs and the uptake of ferritin through *TFRC* or T-cell immunoglobulin mucin domain 1 protein (Tim-1). The release of iron in the brain extracellular space may also be obtained through the release of extracellular vesicles from ECs, transporting *TF*- and ferritin-bound iron [13].

Brain iron status most probably regulates iron release in the extracellular space by ECs at the BBB. As stated above, most brain *TF* derives from oligodendrocytes and epithelial cells of the choroid plexus. However, brain cells that uptake iron through the endocytosis of the holo-*TF*/*TFRC* complex also release apo-*TF* by exocytosis. Brain apo- and holo-*TF* levels may regulate iron uptake through ECs, respectively, by increasing *FPN1/SLC40A1* stability and *HEPH* activity within the abluminal membranes of ECs or decreasing *FPN1/SLC40A1* levels. *HAMP* expressed by astrocytes and the choroid plexus may play a further regulative role in brain iron uptake through the BBB [13].

As depicted above, astrocytes are peculiar cells whose role in brain iron homeostasis is to minimize iron toxicity and export iron in a controlled way. These cells acquire iron released by ECs through *DMT1/SLC11A2*, that is highly expressed in the astrocyte endfeet, directly contacting ECs. Small molecules like hydrogen ions, citrate or ATP, released from astrocytes, may mediate iron delivery from ECs to *DMT1/SLC11A2* on the plasma membrane of astrocytes. Iron is then released into the extracellular space through *FPN1/SLC40A1* coupled with *CP*. Astrocytes are mostly involved in iron trafficking while not in brain iron accumulation, although these cells may accumulate iron with age. Oligodendrocytes need high amounts of iron for their functions during their proliferation and differentiation and to produce myelin. These cells acquire iron for their substantial necessities by the classical holo-*TF*/*TFRC* complex during oligodendrocyte maturation and by *FTH1* uptake through a specific receptor that has been identified in mice as the T-cell immunoglobulin mucin domain 2 protein (*Tim-2*) and probably as Tim-1 in humans. Mature oligodendrocytes do not express *TFRC* while mainly acquiring iron through *FTH1* uptake, which is probably mediated by Tim-1 [14]. Ferritin iron storage is particularly important in these brain cells, since they handle large amounts of this potentially dangerous metal. Oligodendrocytes also express *FPN1/SLC40A1*, suggesting that iron may be released through this channel also in these cells. Brain resident microglia cells originate from yolk sac macrophages during embryogenesis. These cells acquire iron by endocytosis of the holo-*TF*/*TFRC* complex and by *DMT1/SLC11A2,* store iron excess in the ferritin shell and export iron through *FPN1/SLC40A1*. Microglia may also release ferritin that may be acquired by oligodendrocytes for their iron needs. Iron enters neurons through the *TF-TFRC* mediated process and through *DMT1/SLC11A2.* Iron released in the cytosol can be used, stored by ferritin or released by *FPN1/SLC40A1*. The regulation of brain iron homeostasis in all brain cells is orchestrated by IRPs, being *IRP2* the main sensor of cytosolic iron levels [15]. Although the main routes of iron handling in the brain are known (Figure 4), there are still several gaps to be clarified in the homeostasis of this necessary but harmful metal that is implicated in several and different neurological disorders.

## 3. Iron and FTD: What Is Currently Known Based on Clinical, Imaging, Histological and Biochemical Studies

Iron levels in the brain increase during the first 30 years of life in response to the stringent metabolic needs; then, the brain iron content reaches a plateau for a further 30 years and returns to increase gradually with aging. During healthy aging, iron accumulates in particular regions of the CNS, mainly the basal ganglia and areas related to motor control, which is probably due to dysfunctions or increased permeability of the BBB, chronic neuroinflammation or age-related changes in iron homeostasis and distribution. Nonetheless, in healthy aging, iron is maintained in a safe form in the CNS, which is carefully held under strict control, and it is mainly bound to neuromelanin and ferritin [6]. Neurodegenerative disorders, and in particular dementias, mainly appear with aging; then, aging may be considered the first risk factor for this type of diseases. Since brain iron levels increase with aging, many studies have investigated the possible link between this increase and neurodegeneration. Dysfunctions of iron homeostasis are indeed observed in several neurodegenerative disorders, although it is still challenging to determine whether iron dyshomeostasis must be considered a direct cause of neurodegeneration, a contributor factor, simply a consequence of other neurodegenerative mechanisms or a completely unrelated event [16]. The genetic manipulation of iron-related genes in different animal models resulted in rescue of neurodegeneration in some genetic disorders. The use of chelating agents in animal models gave also interesting results in some neurodegenerative disorders, showing neuroprotection, and suggesting a substantial contribution of iron dyshomeostasis in neurodegeneration. However, clinical trials with chelating agents in humans gave often inconclusive results. Further research is then needed to define if iron dyshomeostasis can be considered a cause or a consequence of neurodegeneration [16].

In neurodegenerative disorders, iron may accumulate through several mechanisms, like mitochondrial dysfunctions, protein misfolding and aggregation, failure of the autophagic–lysosomal degradative pathways of iron-bound proteins or neuroinflammation, and they may contribute to neuron loss through different cell death mechanisms, like apoptosis or more directly through ferroptosis [17,18,19]. Iron dyshomeostasis and accumulation in the CNS have been thoroughly described in the most common and investigated neurodegenerative disorders like AD, PD, and Huntington’s disease (HD), but also in less prevalent disorders like Friedreich’s ataxia (FRDA) or NBIAs, and we refer the readers to excellent reviews on these diseases [20,21,22,23,24]. However, iron metabolism dysfunctions are also featured in neurodegenerative disorders strictly interconnected with FTD.

ALS shares several clear overlapping features with FTD from clinical traits to pathogenetic pathways and genetics. Iron accumulation in the motor cortex is a neuropathological hallmark in ALS, which is clearly evidenced by several neuroimaging studies [25]. Evidence of iron homeostasis disruption in spinal cord tissues of ALS patients and ALS animal models, with increased iron and ferritin content accompanied by microgliosis, has been thoroughly described [26,27,28]. Ferritin was also found to be increased in the serum of ALS patients, although it is currently not clear if these findings are related to survival [29,30]. Of note, the H63D variant in the iron-related HFE gene is considered a genetic risk factor increasing susceptibility to ALS and accelerating disease progression in animal models [31].

As depicted above, FTLD comprises the extrapyramidal atypical parkinsonian syndromes PSP and CBS, which are classified in terms of pathomechanisms as tauopathies [1,32]. Iron has already been implicated in these and further tauopathies, like AD and Niemann–Pick’s disease [33,34,35]. PSP is a primary tauopathy; i.e., *MAPT/TAU* pathology is the main driver of neurodegeneration. Brain iron accumulation has been demonstrated by neuroimaging studies in PSP and other atypical parkinsonian syndromes like multiple system atrophy (MSA) [36,37]. *MAPT/TAU* filaments co-localize with ferritin in PSP brains, particularly in glial cell [38], but astrocytes may also accumulate iron [39,40]. Neuroimaging studies also revealed brain iron accumulation in CBS with different patterns in comparison with PSP [36,41]. As depicted more in detail below, several studies also demonstrated the possible role of iron in inducing *MAPT/TAU* aggregation in several tauopathies.

Given the involvement of iron homeostasis disruption in several neurodegenerative disorders like ALS and the clinical overlap of FTD with other forms of FTLD like PSP and CBS, it may be stimulating to hypothesize that iron dyshomeostasis could be involved even in FTD, i.e., bv-FTD, sv-PPA and nfv-PPA. Nonetheless, only in the most recent years the possible role of iron in FTD has been consistently considered and evaluated.

Increased iron content was first reported in 1984 in the post-mortem brain of two patients with Pick’s disease [42]. Later, in 1996, immunohistochemistry analysis on the frontal and temporal cortex from clinically defined FTD cases revealed severe microglial activity defined by high ferritin immunoreactivity [43]. Further evidence of the involvement of iron in FTD pathology derived from a proteomic analysis on the human frontal cortex of FTD cases harboring the P301L and the intronic E10+3 variants in the MAPT/TAU gene. This analysis revealed aberrantly regulated proteins in the frontal cortex, including FTL that was found to be upregulated [44].

The clinical overlapping between NBIA and FTD is among the first suggestions of a possible role of iron in the FTLD spectrum disorders. Cognitive decline with dementia and psychiatric and behavioral symptoms resembling FTD have been described in adult cases with NBIA [45,46]. In 2009, Santillo et al. [47] described a case of late-onset NBIA presenting as FTD with ALS in a 70-year-old man with a family history of dementia, AD, FTD and ALS. A first diagnosis of NBIA was based on the classical “eye of the tiger” sign at Magnetic Resonance Imaging (MRI) analysis, with iron deposits in the globus pallidus, but no pathogenic mutations were found in the main genes involved in the pathology that were at least known at that time (pantothenate kinase2, PANK2; phospholipase A2 group VI, PLA2G6 and FTL). The patient’s clinical history further revealed changes in personality with progressive disinhibition, emotional blunting, loss of motivation, impulsiveness, socially inappropriate behavior, diminished empathy, and worsened memory. Fluorodeoxyglucose positron emission tomography (18F-FDG PET) demonstrated hypometabolism in the frontal and temporal lobes, fulfilling the criteria for FTD diagnosis. FTD symptoms were also accompanied by gait difficulties, dystonia, effortful swallowing and dysarthric pseudobulbar speech, although neurophysiology did not suggest the diagnosis of ALS. Given the family history, further genetic analyses of the proband and his siblings affected by FTD and AD were performed, but no pathogenic mutations were identified in superoxide dismutase 1 (SOD1), GRN, MAPT/TAU (exons 9 to 13), synuclein alpha (SNCA), amyloid beta precursor protein (APP) (exons 16 to 17) and presenilin 1 (PSEN1) genes. Further cases of overlap between NBIA and FTD have been described in the literature, which included an adult case of NBIA characterized by the presence of iron deposits in the globus pallidus and substantia nigra and the coexistence of *TARDB/TDP-43* and *MAPT/TAU* pathology [48].

More recent and strong evidence of the role of iron in FTD derives from MRI studies. De Reuck et al. [49] performed an MRI analysis on post-mortem brains of patients affected by distinct neurodegenerative diseases like FTLD, AD, ALS, PSP, DLB and vascular dementia. For the first time, they found an increased iron load in the claustrum, caudate nucleus, putamen, globus pallidus, thalamus and subthalamic nucleus only in FTLD cases with higher metal content in the FTLD-FUS and FTLD-TDP in comparison with FTLD-tau cases. Combining MRI and histopathological examinations, the same authors later confirmed increased iron deposition in the basal ganglia of FTLD cases, particularly those with *FUS* and *TARDBP/TDP43* pathology, also showing increased cortical micro-bleeds in the frontal and temporal lobes, suggesting a possible influence of vascular risk factors on iron accumulation [50]. By MRI analysis, we further showed iron deposition and atrophy in the basal ganglia of FTLD cases harboring the H63D variant in the iron-related HFE gene. Iron deposition was also accompanied by more severe behavioral disturbances, suggesting that HFE could represent a disease-modifying gene in FTLD, while further corroborating the hypothesis of a crucial role of iron in FTLD pathology [51]. By susceptibility-weighted imaging (SWI), Sheelakumari et al. [52] further demonstrated increased iron levels in bilateral superior frontal and temporal gyri, anterior cingulate, putamen, right precentral, right insula, right hippocampus and right red nucleus in patients with bv-FTD compared with control subjects, with a positive association between apathy and iron content in the superior frontal gyrus and between disinhibition and iron content in the putamen. They also found increased iron in the left superior temporal gyrus in patients with PPA, also suggesting that right superior frontal gyrus iron deposition may discriminate between bv-FTD and PPA. A joint ex vivo MRI and histopathology study found iron-rich superficial cortical layer astrocytic processes surrounding small blood vessels with little involvement of the adjacent white matter and dystrophic patterns of punctate iron-rich microglia across the gray matter in FTLD-TDP sporadic cases. In contrast, FTLD-tau sporadic cases showed iron-positive ameboid and hypertrophic microglia and astrocytes in deeper gray matter and adjacent white matter, suggesting distinct mechanisms of neuroinflammation in FTLDs with distinct pathologies [53]. Still, by combining histopathological and MRI analyses, Giannini et al. [54] recently focused on genetically defined FTD cases, considering both FTD-tau cases due to MAPT/TAU mutations and FTD-TDP cases linked to the hexanucleotide large expansion mutation in the C9orf72 gene. Histopathological analyses demonstrated cortical iron accumulation both in MAPT/TAU-FTD and to a smaller extent in C9orf72-FTD cases with similar diffuse distribution in the two genetic groups. Importantly, cortical iron accumulation was observed in activated and dystrophic microglia and reactive astrocytes and associated with the severity of proteinopathy and neurodegeneration. The authors also observed a good correspondence between changes in cortical iron distribution showed by histopathological examinations and hypointense cortical abnormalities seen by ultrahigh field T2*-weighted ex vivo MRI, suggesting in vivo iron imaging as a non-invasive marker to identify neuroinflammation and pathology in FTLD cases.

As described above, iron involvement has been thoroughly studied in ALS and ALS cases often presented also with FTD symptoms. A recent report described the case of a patient with ALS who presented with speech apraxia as early symptom, which was accompanied by upper motor neuron deficiencies. The patient presented with severe *TARDBP/TDP43* pathology and focal iron accumulation in the precentral gyrus and frontal operculum [55]. The same authors interestingly previously reported that the knockdown of the Tardbp/Tdp43 gene in mice resulted in impaired axonal transport of several ribosomal protein mRNAs but also of ferritin mRNA in cortical neurons, suggesting that *Tardbp/Tdp43* may affect iron homeostasis in mice neurons. A more recent report, currently published only as a preprint (not yet peer-reviewed) manuscript in bioRxiv [56], interestingly reported the association of amygdala intra-neuronal phosphorylated *TARDBP/TDP43*, cytoplasmic *TARDBP/TDP43* pathology and ferritin levels with behavioral symptoms in sporadic ALS, further suggesting a role of *TARDBP/TDP43* in regulating iron homeostasis and modulating behavioral symptoms in ALS patients.

A recent study analyzed the serum profile of bv-FTD and PPA patients relative to Fe- and Cu-related biomarkers [57]. The authors found no significant differences in comparison with control subjects in any serological investigated marker (iron, *TF*, *FTL*, *TF* saturation, total iron binding capacity, *CP*, *CP* enzymatic activity, *CP*/*TF* ratio) except for *CP* enzymatic activity/*CP* content ratio only in PPA patients, being significatively decreased. Although iron-related parameters were found unchanged, the authors speculated that the decrease functionality of *CP* may suggest potential anomalies in iron handling in PPA, considering the ferroxidase activity of *CP* and its role in iron cellular export. No difference in total iron concentration in the CSF was also recently found in bv-FTD cases compared to control subjects [58].

Finally, a recent study [59] analyzed the role of genetic variants related to the immune system and inflammation in modulating AD and FTLD. The authors considered polymorphisms in 50 genes involved in the selected pathways, analyzing sporadic AD and FTLD cases and carriers of GRN and C9orf72 mutations. Among the 50 selected genes, the TF and aconitase 2 (ACO2) iron-related genes were also considered. By linear regression analyses, the authors found that the rs1049296 polymorphism in the TF gene was associated with age at onset in the sporadic AD + FTLD group, but they concluded that the association was driven by the sporadic AD group only. The same TF polymorphism was previously reported to be a risk factor for AD in synergy with the C282Y (rs1800562) allele of the HFE gene, although, by using a larger dataset, the association between the two polymorphisms in the TF and HFE genes and AD risk was not furtherly confirmed [60,61].

In conclusion, evidence of dysfunctions in brain iron homeostasis in clinical, imaging and biochemical studies in FTD is still scarce; nonetheless, the recent literature (summarized in Table 1) strongly suggests further research in this area.

In the next section, we review and emphasize further studies demonstrating a direct or close connection between iron homeostasis and genes associated with FTD or strictly linked with FTD-associated genes, although not always or not necessarily currently considered in the perspective of FTD, but that we suggest would be taken into account in future research, with a view to increase the knowledge in FTD pathomechanisms.

## 4. FTD-Associated Genes and Iron Homeostasis

In this section, we start briefly describing the main known neurodegenerative disorders caused by mutations in genes directly involved in iron homeostasis and handling to draw the reader’s attention to the pathological mechanisms that dysfunctions strictly linked to iron metabolism may trigger. We then describe further research regarding the proven role in iron homeostasis of genes directly involved in genetic forms of FTD or engaged in pathways strictly related to FTD-associated genes with the aim to foster future research.

### 4.1. Neurodegenerative Disorders Caused by Variants in Genes Directly Involved in Iron Handling

Variants in a few genes directly involved in iron homeostasis have been associated with neurodegenerative diseases [62]. NBIA comprises a clinically and genetically heterogeneous group of rare neurodegenerative disorders, which are all characterized by the degeneration and accumulation of iron in the basal ganglia, particularly the globus pallidus and substantia nigra [23]. Only two NBIA disorders are caused by mutations in genes expressing proteins definitely and directly involved in iron homeostasis, namely FTL and CP, causing, respectively, neuroferritinopathy and aceruloplasminemia. Both disorders are characterized by a more pronounced iron accumulation in the iron-rich regions of the brain in comparison to other NBIAs. Neuroferritinopathy is an autosomal dominant NBIA, characterized by adult-onset movement symptoms like chorea, parkinsonism and dystonia, with also frequently observed cognitive and behavioral defects. In this disorder, intranuclear and intracytoplasmic bodies containing ferritin and insoluble iron have been found in glial cells and neurons and in other body cells [63]. The most frequent mutations associated with this NBIA indeed result in the decreased ability of ferritin to incorporate iron. This defect results in both the aggregation and intracellular precipitation of instable ferritin and the increase in the cellular free iron pool. As a consequence, oxidative stress is increased [64,65]. Aceruloplasminemia, an autosomal recessive adulthood NBIA, is a further movement disorder also characterized by cognitive dysfunctions, retinopathy, microcytic anemia and diabetes mellitus [66]. Mutations in the CP gene result in structural modifications of the expressed protein that cause its defective ferroxidase activity. In the CNS, mainly in astrocytes, this defect turns out in the cellular uptake of an excess of ferrous iron that cannot be oxidized and cannot also be exported by *FPN1/SLC40A1* in the absence of a functional *CP*. Astrocytes then accumulate ferrous iron, while neurons become iron-deficient and die [67,68,69]. Further genes associated with other forms of NBIAs are not directly involved in iron homeostasis as iron-handling proteins; nonetheless, their mutations may heavily affect iron homeostasis in the CNS [23]. As an example, mutations in the WD repeat domain 45 (WDR45) gene, specifying the autophagy protein WD repeat domain phosphoinositide-interacting protein 4 (*WIPI4*), may result in impaired ferritinophagy, which is the main pathway involved in iron release from cytosolic ferritin [70]. It is worth noting that as stated above, some cases of NBIA were found to present with symptoms resembling FTD [45,46,47].

Further rare neurodegenerative diseases are determined by mutations in genes coding for proteins that belong to the cellular machinery involved in the synthesis of ISCs and/or their transfer to ISC-containing proteins. ISCs are crucial redox-active co-factors of several mitochondrial, nuclear and cytoplasmic proteins that mediate reactions within the electron transport chain, citric acid cycle, heme biosynthesis, fatty acid oxidation, RNA metabolism, t-RNA modifications, DNA replication and repair, cell cycle, protein translation, translational regulation and iron homeostasis [71]. Multiple mitochondrial dysfunctions syndrome (MMDS) is a rare recessive disorder with onset in early infancy, which is characterized by severe brain dysfunctions and psychomotor delay. The syndrome is caused by mutations in genes that play an essential role in the biogenesis of mitochondrial ISC-containing proteins, like NFU1 iron-sulfur cluster scaffold (NFU1), bola family member 3 (BOLA3), iron sulfur cluster assembly factor IBA57 (IBA57), iron sulfur cluster assembly 2 (ISCA2), and iron sulfur cluster assembly 1 (ISCA1) [72]. FRDA is a further neurodegenerative disorder due to mutations of a gene involved in ISC biogenesis and the most common among hereditary ataxias. It is characterized by early onset, slowly progressive ataxia with areflexia, dysarthria, scoliosis, muscular weakness and wasting, and it is associated with hypertrophic cardiomyopathy and diabetes mellitus. FRDA is an autosomal recessively inherited disorder that is mainly associated with the homozygous GAA-triplet expansion in the first intron of the frataxin (FTX) gene [73]. Other genes of the ISC machinery are involved in further very rare neurological diseases [74].

A further interesting iron-related gene, whose mutations have only recently been associated with neurodegenerative disorders, is IRP2/IREB2, expressing an RNA-binding protein that crucially regulates the translation and stability of several mRNAs for iron-related proteins. Irp2/Ireb2 gene deletion in mice causes a late-onset movement disorder characterized by lower motor neuronal degeneration with significant spinal cord axonopathy, progressive loss of motor capabilities, ataxia, bradykinesia and tremor that progress slowly as the animals age. Neurodegeneration is preceded by abnormal iron metabolism in the brain and spinal cord and is also accompanied by a mild form of anemia [75,76,77,78]. More recently, a more extensive behavioral testing of Irp2-null mice highlighted a significant motor deficit with also compromised somatosensory functions and cognitive impairment related to prefrontal cortex dysfunctions [79], which is a region of the brain that is affected in FTD [80,81]. Only three patients with biallelic variants in the IRP2/IREB2 gene have been described to date. The first described patient was a 16-year-old male with a biallelic loss of function variants of the gene and a choreoathetoid movement disorder with microcytic hypochromic anemia unresponsive to iron supplementation [82]. A second case was a child with a missense and an in-frame deletion variant in IRP2/IREB2 gene, who died at 10 years of age because of a progressive neurological disease characterized by profound development delay, mild dystonia and athetosis, which was also accompanied by neutropenia and mild microcytic anemia [83]. The third, a 7-year-old patient, was compound heterozygous for biallelic missense IRP2/IREB2 mutations and developed a disease characterized by profound neurodevelopment delay, seizures, dystonia and choreiform movements [84].

Two further proteins, whose corresponding genes are strictly involved in neurodegenerative disorders with higher prevalence, like AD and PD, have also a role in maintaining iron homeostasis. *APP* has a ferroxidase activity like *CP*, facilitating cellular iron export through *FPN1/SLC40A1*, while *SNCA* is also a cytosolic ferrireductase. The transcripts of APP and SNCA genes contain an IRE-like sequence in their 5′-UTR; then, their translation is directly regulated by iron like for several iron-related genes [85]. Interestingly, iron can also increase *APP* amyloidogenic processing [86], while iron binding to *SNCA* leads to its oxidation, unfolding and aggregation [87], as we will further discuss below.

The number of genes directly involved in CNS iron homeostasis and neurodegenerative disorders is likely to increase in the future. An interesting recent work performed a systematic bioinformatic analysis, integrating data obtained in induced pluripotent stem cell (iPSC)-derived neurons and derived from a genome-wide clustered regularly interspaced short palindromic repeats interference (CRISPRi) phenotypic screening for genes involved in iron accumulation with CRISPR-based functional genomics studies with the aim to identify new genes involved in the maintenance of neuronal iron homeostasis [88]. The authors found that dysfunctional mitochondrial electron transport chain, impaired macroautophagy and lysosomal and retromer complex pathways are the main contributors to neuronal iron dyshomeostasis, as expected, but interestingly expanding the number of involved genes and revealing previously poorly characterized proteins causing this phenomenon. They also newly observed that the perturbed synthesis of glycophosphatidylinositol (GPI) and trafficking of GPI-anchored proteins trigger neuronal iron accumulation in a cell-autonomous manner. Then, in the future, the analyses of large numbers of functional genomics data will probably increase the number of genes involved to some extent in iron homeostasis and handling in the CNS, and that may be implicated in genetically determined neurodegenerative disorders.

### 4.2. Evidence of Interconnections Between FTD-Associated Genes and Iron Homeostasis: Old and New Perspectives

Only a few genes whose variants are associated with FTD have also been implicated, although mostly not directly involved, in iron homeostasis. In this section, we describe currently known links between FTD-associated genes and iron metabolism and suggest further evidence of the possible interconnections between iron pathways and FTD pathology, which would deserve future deepening research.

Mutations in the coiled-coil–helix–coiled-coil–helix domain containing 10 (CHCHD10) gene were first involved in FTD and ALS in 2014 [89]. *CHCHD10* is a mitochondrial protein localized in the intermembrane space, enriched in the cristae junctions and involved in the maintenance of the mitochondrial network and cristae morphology. R15L and S59L mutations associated with FTD have been shown to induce mitochondrial damage and the cytosolic accumulation of *TARDBP/TDP43* [90]. Murine knock-in models of the FTD-associated S59L mutation show aberrant mitochondrial morphology and function with mitochondrial myopathy and spinal MND. Brain regions initially seemed to be only mildly affected, and mice showed no signs of cognitive decline [91,92]. A later more comprehensive behavioral, electrophysiological and neuropathological assessment of one knock-in model demonstrated that it also reproduces the signs and symptoms of FTD, with memory impairment and anxiety-related behavior disturbance, related to hippocampal protein aggregation, gliosis and neuronal degeneration [93]. Transgenic mice models of the same R15L and S59L mutations show impaired mitochondrial fusion and respiration in the brain, and such changes have also been found in the brains of FTLD-TDP patients and cellular and animal models of *TARDBP/TDP43* pathology [94]. A further study demonstrated that insoluble *CHCHD10* aggregates accumulate and co-localize with phospho-*TARDBP/TDP43* inclusions in the frontal cortex of FTLD-TDP. Transgenic mice expressing the R15L and S59L mutations in the CNS also show *CHCHD10* insoluble aggregates and *TARDBP/TDP43* pathology in the frontal cortex neurons [95]. In in vivo and in vitro models, the same mutations have been shown to impair the mitophagy flux, which was an observation that was confirmed in human FTD brain tissue [96]. A recent study considering a further FTD-associated CHCHD10 mutation (V57E) confirmed the induction of mitochondrial dysfunctions, showing that the variant alters the structure of the protein, inducing the increase in mitochondrial superoxides and impairing mitochondrial respiration [97]. Overall, all the above studies pointed out severe deficiencies in mitochondrial functions related to FTD-associated CHCHD10 mutations. As further discussed in this section, mitochondrial dysfunction is a common finding in FTD as well as in several further neurodegenerative disorders [98]. Mitochondria are the cellular site where iron is mainly used, most proteins of the respiratory chain having iron as a co-factor. Mitochondria are also deeply involved in iron homeostasis, being the main site of the synthesis of heme and ISCs. Then, it is not surprising that mutations in genes associated with FTD and altering mitochondrial functions may affect iron homeostasis. Interestingly, the silencing of the CHCHD10 gene in HEK293 cells was indeed demonstrated to alter iron homeostasis, increasing iron mitochondrial content and decreasing iron cellular efflux while maintaining iron cytosolic levels. These findings indicate that the CHCHD10 gene may be involved in regulating iron transport in mitochondria although not affecting the oxygen consumption rate or mitochondrial ATP synthesis. In contrast, however, disease-associated mutants expressed in HEK293 cells induced only protein mislocalization and mild bioenergetic defects [99]. The experimental evidence that the CHCHD10 gene may be involved in the regulation of mitochondrial iron would merit further investigations in FTD, considering the effects of human CHCHD10 mutations on mitochondrial functions and the central role of the organelle in iron homeostasis [99].

Charged multivesicular body protein 2B (CHMP2B) is a further gene involved in rare early-onset autosomal dominant FTD forms (FTD type 3), which are characterized by personality and behavior changes and *MAPT/TAU*, *TARDBP/TDP43* and *FUS* negative but ubiquitin and/or sequestosome 1/protein 62 (*SQSTM1/p62*) positive inclusions. The first disease causing mutation in this gene was identified in the splice acceptor site of its last exon, resulting in aberrant mRNA splicing and the production of two novel transcripts, whose translation leads to the formation of two C-terminally truncated proteins [100]. The *CHMPB2* protein is an essential component of the endosomal sorting complex required for transport III (ESCRT-III), playing a crucial role in membrane scission events and endo-lysosomal and autophagy functions [101]. The endo-lysosomal and autophagy pathways are strictly linked to iron homeostasis through the specialized pathways that regulate the release of iron from the ferritin shell. Noteworthy, Zhang et al. [102] interestingly observed that iPSC-derived forebrain cortical neurons obtained from FTD patients harboring the CHMP2B splicing mutation also displayed aberrant mitochondria morphology with the severe impairment of cristae formation and preferential perinuclear localization, impaired mitochondrial functions with reduced basal respiration and maximal capacity, and increased oxidative stress. RNA-seq analyses in these cellular models interestingly revealed an imbalance of iron homeostasis with the mis-expression of several genes involved in iron homeostasis, which was accompanied by increased cytoplasmic Fe^2+^ levels [102]. These findings suggest the link between the disruption of endo-lysosomal/autophagy pathways and iron dyshomeostasis for future studies on FTD pathomechanisms, considering that multiple FTD-associated genes play a role in the endo-lysosomal system and autophagy pathways [103,104].

An interesting link between iron homeostasis and the C9orf72 gene has been firstly hypothesized by the observation that the 5′-UTR of the gene transcript, immediately before the start codon, shows an IRE-like structure, similarly to the 5′-UTR IRE structure of ACO2, FTH1, FTL and other iron-related genes strictly linked to neurodegenerative disorders like APP and SNCA [105]. An interesting paper also showed that the G-quadruplex structures formed by both RNA and DNA (G_4_C_2_)_4_ hexanucleotide repeats, characteristic of the C9orf72 intronic expansions, bind heme and enhance its intrinsic peroxidase and oxidase activity under physiologically plausible environmental conditions, suggesting both the sequestration of heme from fundamental iron-related cellular functions like mitochondrial respiration and the catalysis of oxidative reactions that may enhance neuronal oxidative damage [106]. Furtherly, as already cited above, FTD patients harboring the C9orf72 hexanucleotide expansion show cortical iron accumulation in activated and dystrophic microglia and reactive astrocytes that correlate with the severity of neurodegeneration [54], further suggesting a possible role of *C9orf72* in disrupting iron homeostasis.

The genes C9orf72 and transmembrane protein 173/stimulator of interferon genes (TMEM173/STING) are strictly interrelated. Myeloid cells from the C9orf72 knockout mice and neurons of ALS and FTD models of C9orf72 expansion show the hyperactivation of the *STING* pathway with increased type I interferon signature [107,108]. The *STING* pathway activation was previously attributed to the disruption of the *STING* lysosomal/autophagic degradation caused by the lack of *C9orf72* [108], which is known to be involved in the lysosome–autophagy pathway [109]. The same activation was, however, seen in human iPSC-derived neurons harboring not only the C9orf72 hexanucleotide expansion but also further different familial ALS-causing mutations in genes like TARDBP/TDP43, profilin 1 (PFN1), FUS, kinesin family member 5A (KIF5A), and NIMA related kinase 1 (NEK1), and it was associated with DNA damage [107]: a pathway that is indeed increasingly implicated in ALS but also in FTD [110]. Particularly, mitochondrial dysfunction and subsequent mitochondrial DNA damage have been implicated in both ALS and FTD [93,111,112,113,114]. It is then tempting to speculate that nuclear, and particularly mitochondrial DNA damage, may be the factors driving *STING* activation in ALS and FTD. Although not investigated in FTD or other neurodegenerative disorders to date, cellular iron overload might also be the direct trigger of *STING* activation in neurons and/or further brain cells like microglia and astrocytes. Indeed, iron overload has been shown to enhance the activation of the *STING* pathway in HepG2 cells and mice liver [115]. *FTX* haploinsufficiency causes mitochondrial iron overload, and it is particularly deleterious for neurons and cardiomyocytes, which are the most involved cells in the pathogenesis of FRDA. Its knockdown in cardiomyocytes derived from iPSCs and further cellular models has been demonstrated to result in the activation of the *STING* pathway, which was only partly explained by mitochondrial DNA release in the cytoplasm [116]. A further mechanistic link between iron overload and *STING* activation has been demonstrated in chondrocytes using an in vivo model of hemophilic arthropathy [117]. Iron overload and/or dyshomeostasis may also indirectly trigger the activation of the *STING* pathway and cellular senescence through the subsequent induction of mitochondrial dysfunction and mitochondrial DNA release, underlining the close connection among iron dyshomeostasis, mitochondrial DNA release and *STING* activation [118]. On the converse, *STING* activation in the brain has been found to induce iron overload by enhancing ferritinophagy in a murine model of ischemia–reperfusion injury [119] and to cause liver iron accumulation in a murine model of acute autoimmune hepatitis [120]. Considering that *STING* activation has been observed in human iPSC-derived neurons harboring the C9orf72 hexanucleotide expansion [107] and that cortical iron accumulation has been demonstrated in C9orf72-FTD cases [54], it is then tempting to speculate that brain iron overload associated with the C9orf72 mutation might be indirectly induced by the *STING* activation derived by the C9orf72 mutation itself. In conclusion, the above experimental evidence more generally suggests that further research is needed to better elucidate the interconnection between the *STING* pathway and iron homeostasis in human diseases, particularly in neurodegenerative disorders with neuroinflammation and mitochondrial involvement like FTD.

Neurons are particularly susceptible to mitochondrial defects, since they are non-dividing post-mitotic cells that need high energy amounts for their functions and an efficient machinery for mitochondrial dynamics regulation. A huge amount of experimental evidence emphasizes mitochondrial dysfunctions as emerging hallmarks in FTD [113,114,121,122,123,124,125,126,127,128,129], and mitochondrion is the main site of cellular iron handling. A recent study highlighted the crucial role of mitochondrial defects in *Drosophila* models of C9orf72 ALS/FTD, suggesting that mitochondrial oxidative stress is a relevant mechanistic contributor to C9orf72 pathogenesis, leading to mitochondrial dysfunction and indicating the *KEAP1/NRF2* signaling pathway as a key therapeutic target [130]. As described above, *NRF2* is a master regulatory transcription factor for antioxidant cellular response that, under oxidative stress conditions, binds AREs in the promoter region of several genes, some of which are also involved in iron homeostasis, like FTL, FTH1 and FPN1/SLC40A1, regulating their transcription in order to avoid iron-induced cellular damage [7,12].

SQSTM1/p62 is another FTD-associated gene. *SQSTM1/p62* protein has a role as a receptor for selective autophagy and can also shuttle polyubiquitinated substrates for degradation via the proteasome [131]. Notably, the SQSTM1/p62 gene holds an ARE element in its promoter and is one of the genes transcriptionally regulated by *NRF2* [132], together with several genes involved in iron homeostasis. At the same time, *SQSTM1/p62* competitively binds to *KEAP1* through its *KEAP1*-interacting region (KIR) to activate *NRF2*, creating a positive feedback loop that further contributes to the transcriptional activation by *NRF2* as a response to oxidative stress [133]. Several ALS- and FTD-associated mutations in the SQSTM1/p62 gene have been found to impair the interaction of *SQSTM1/p62* with *KEAP1*, in turn decreasing the *NRF2* antioxidant transcriptional response [132,134,135,136]. Notably, the ALS- and FTD-associated gene TANK binding kinase 1 (TBK1) is involved in the phosphorylation of *SQSTM1/p62*, and mutations in this gene may affect *NRF2* signaling [136]. As already mentioned, in principle, the inactivation of *NRF2* can also modify the expression of iron-related genes, in turn possibly altering iron homeostasis. This hypothesis deserves future research to be elucidated in FTD.

Several FTD-linked mutations in TARDBP/TDP43, FUS, C9orf72 and MAPT/TAU genes have also been shown to disrupt endoplasmic reticulum (ER)–mitochondria communications [137]. ER membranes are recruited to mitochondria by scaffolding proteins that function to tether the two organelles and indeed are named “tethering proteins”. Among these proteins, the integral ER protein vesicle-associated membrane protein associated protein B (*VAPB*) and the outer mitochondrial membrane protein tyrosine phosphatase interacting protein 51 (*PTPIP51*) play a crucial role in communications, regulating Ca^2+^ exchange between the two organelles, mitochondrial ATP production and synaptic activity. Mutations in TARDBP/TDP43 and FUS have been shown to activate the glycogen synthase kinase-3β (*GSK3β*), resulting in the decreased binding of *VAPB* to *PTPIP51* and disrupted ER–mitochondria signaling [137]. Interestingly, *GSK-3β* is also known to modulate the cellular response to oxidative stress through the phosphorylation of *NRF2*, resulting in *NRF2* degradation and its nuclear exclusion in a *KEAP1*-independent manner [138]. As already observed, several *NRF2*-controlled genes are intriguingly involved in maintaining iron homeostasis [7].

Several autosomal dominant mutations in the MAPT/TAU gene have been found to cause FTD with parkinsonism linked to chromosome 17, which is characterized by the presence of filamentous *MAPT/TAU* inclusions (FTLD-tau) in the atrophic frontal and temporal lobes. Recently, as described above, iron accumulation has been observed in the frontal and temporal cortices of FTD patients harboring MAPT/TAU mutations, and this accumulation has been found to be associated with pathological severity [54]. Interestingly, Mapt/Tau knockout mice have been found to develop age-dependent brain atrophy and neurodegeneration accompanied by iron accumulation in the cortex, hippocampus and substantia nigra. In murine primary neuronal cultures, the loss of Mapt/Tau was found to induce iron accumulation through the impairment of *APP* trafficking to the cellular membrane and, consequently, through the reduction in its interaction with *FPN1/SLC40A1*, causing the decrease in iron export [139]. This work is the first one, to the best of our knowledge, describing the direct involvement of the Mapt/Tau gene in iron homeostasis. The same research group further confirmed these findings, demonstrating that lithium treatment induces iron accumulation in mice brain and murine primary cortical neuronal cultures due to the decrease in soluble *Mapt/Tau* levels induced by the drug and that lithium-induced neuronal iron accumulation requires the expression of the *Mapt/Tau* protein [140]. One further recent work demonstrated that the overexpression of human full-length MAPT/TAU into the hippocampal CA3 region of C57BL/6 mice and in SHSY-5Y and N2a cells induced the abnormal deposition of iron, although the mechanism implicated in this accumulation was not elucidated in this work [141]. Instead, several further published articles considered the role of iron in *MAPT/TAU* phosphorylation, aggregation and accumulation in tauopathies, as we will describe below.

Furtherly, mutations in the OPTN gene are a rare cause of FTD [142]. The *OPTN* protein is a selective autophagy receptor that binds to polyubiquitinated cargoes, bringing them to autophagosomes, mainly in selective autophagy pathways like aggrephagy, xenophagy and mitophagy [143]. Mutations in the gene that are associated to glaucoma and the knockdown of OPTN in HeLa cells have been demonstrated to cause the disruption of *TF* uptake and *TF/TFRC* endosomal recycling [144], suggesting a crucial role of the autophagy receptor also in cellular iron homeostasis. This is a further link between a gene involved in FTD and iron homeostasis, which to date has not been explored in this neurodegenerative disorder, but it deserves to be deepened. It is worth noting that OPTN mutations described in pure FTD are often frameshift mutations that lead to OPTN haploinsufficiency [142].

Mutations in TBK1 are also found in FTD [142] besides ALS. Intriguingly, a recent work has identified Tax1 Binding protein 1 (*TAX1BP1*) as a functional binding partner for NCOA4, regulating NCOA4-related ferritin degradation through an alternative lysosomal transport pathway and independently of its role as a selective autophagy cargo receptor [145]. Under basal and iron starvation conditions, the unc-51 like autophagy activating kinase 1/2 (*ULK1/2*)-RB1 inducible coiled-coil 1 (*RB1CC1* or *FIP200*) complex enables binding between *NCOA4* and ferritin by regulating the dissociation of *NCOA4* from *TAX1BP1* and Golgi membranes. The *NCOA4*–ferritin complex is then trafficked to lysosomes to allow iron release into the cytosol. Knocking out RB1CC1/FIP200, aggregates of *NCOA4* and *TAX1BP1* have been shown to recruit *TBK1*; under basal and iron starvation conditions, *TBK1* is activated, allowing the release of *NCOA4*, its binding to ferritin and the trafficking of the *NCOA4*–ferritin complex to lysosomes for iron release. The researchers also demonstrated that the activation of *TBK1* regulates the redistribution of autophagy-related 9A (*ATG9A*) to the Golgi membranes, enabling the continued trafficking of ferritin and iron release. Interestingly, the ALS- but also pure FTD-associated TBK1 mutation E696K [142] abrogates *TBK1* protein ability to drive iron release from ferritin [145]. Furtherly, *TBK1* has been found to promote the degradation of BTB and CNC homology 1 (*BACH1*), which is a repressor of the expression of genes involved in iron metabolism [146]. All these findings provide new intriguing insights into the direct role of the FTD gene TBK1 in maintaining cellular iron homeostasis.

Finally, a very recent work showed high expression of the transmembrane protein 106B (TMEM106B) gene in SH-SY5Y cells and the substantia nigra of mice, which were both treated with 1-methyl-4-phenyl-1,2,3,6-tetrahydropyridine (MPTP) or its derivative 1-methyl-4-phenylpyridinium (MPP^+^) to mimic PD. In these PD models, iron accumulation was observed, which was accompanied by motor impairment and the loss of dopaminergic neurons in mice and inflammatory response in both models. All these effects were interestingly attenuated by the knockdown of TMEM106B expression [147]. TMEM106B polymorphisms have been associated with the risk to develop FTLD-TDP; in particular, the minor alleles have a protective effect especially in individuals carrying mutations in the GRN gene. Interestingly, higher or lower TMEM106B mRNA expression was observed in the frontal cortex of subjects carrying, respectively, the more frequent risk allele or the minor protective allele in the homozygous state [148]. Further, the protective minor TMEM106B alleles have been associated with higher *GRN* plasma levels [149] and higher GRN mRNA expression [150]. Given the known role of both iron and *GRN* in brain inflammation and in the light of the above-described reduction in iron accumulation and neuroinflammation resulting from TMEM106B knockdown, it would be extremely interesting to assess the brain iron status in individuals carrying the risk and the protective alleles of TMEM106B gene.

In conclusion, several pieces of evidence suggest that at least some FTD-associated genes may be directly or indirectly involved in the maintenance of iron homeostasis (summarized in Table 2). Although not thoroughly considered in currently published research studies regarding FTD pathomechanisms, further studies regarding the role of FTD-associated genes in iron metabolism are then required.

## 5. Further Perspectives for Future Research

### 5.1. Iron Homeostasis and Mitochondrial Dysfunctions

In this section of the review, we focus on the link between iron homeostasis and some crucial mechanisms of neurodegeneration that, in our opinion, would be further deepened in the case of FTD. Mitochondrial dysfunction may be one of these mechanisms. Mitochondrial dysfunction is indeed often observed in FTD patients’ tissues and animal models of the disease [113,114,121,122,123,124,125,126,127,128,129,151], as we thoroughly emphasized in many points of this review. Mitochondria are the cellular organelle where iron is mainly handled. Then, it may be speculated that mitochondrial dysfunctions may affect iron homeostasis and, conversely, iron dyshomeostasis may impact mitochondrial functions.

As already described, mutations in the CHCHD10 gene have been shown to induce mitochondrial damage and dysfunction [90,91,92,93,94,96,97,99] and, as above described, the silencing of the gene has been found to regulate iron transport in mitochondria, increasing mitochondrial iron levels while maintaining cytosolic iron levels unchanged [99]. This effect should affect *FTMT* levels, although the authors did not explore this possibility. iPSC-derived forebrain cortical neurons harboring an FTD-associated mutation in the CHMP2B gene have been found to display aberrant mitochondria morphology, impaired mitochondrial functions and the imbalance of iron homeostasis [102].

**Figure 5:**
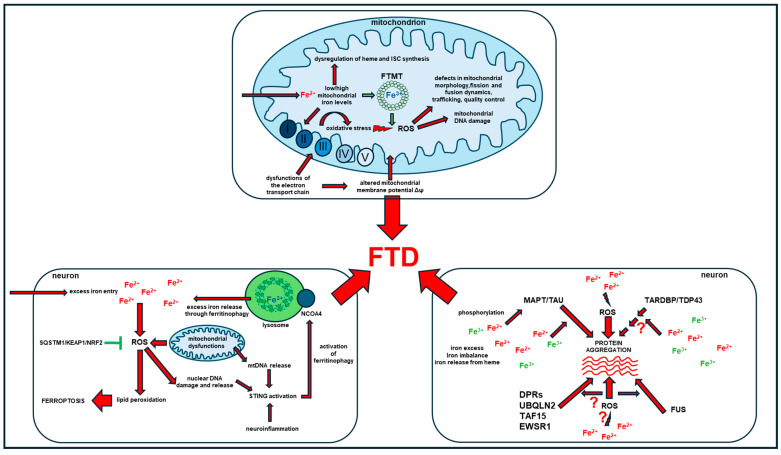
Pathomechanisms of FTD that might involve iron imbalance. Iron metabolism dysfunctions may lead to mitochondrial low iron levels with the possible dysregulation of heme and iron sulfur cluster (ISC) synthesis. A paucity of ISCs and heme may lead to dysfunctions of the electron transport chain and altered mitochondrial membrane potential (Δψ). Together with oxidative phosphorylation impairment, high mitochondrial iron may lead to oxidative stress with the production of excess reactive oxygen species (ROS) that may result in mitochondrial DNA (mtDNA) damage or defects in mitochondrial morphology, fission and fusion dynamics, trafficking and quality control through the mitophagy pathway. Mitochondrial ferritin (*FTMT*) may buffer excess iron and protect against oxidative damage. Excess cellular iron entry or excess release from ferritin through ferritinophagy, together with mitochondrial dysfunctions, may trigger ROS production in neurons, leading to lipid peroxidation and the activation of ferroptotic cellular death. The release of damaged nuclear and mitochondrial DNA due to oxidative stress may trigger, together with neuroinflammation, the activation of stimulator of interferon genes (*STING*) that may exacerbate ferroptosis through the activation of ferritinophagy and further release of harmful iron. The activation of the sequestosome 1/protein 62/kelch-like ECH associated protein 1/NFE2-like bZIP transcription factor 2 (*SQSTM1/p62/KEAP1/NRF2*) signaling pathway, also involved in the regulation of iron-related genes, may inhibit ferroptosis. Iron dyshomeostasis can trigger microtubule-associated protein tau (*MAPT/TAU*) aggregation directly through the generation of ROS or through the modulation of *MAPT/TAU* phosphorylation. Oxidative stress and ROS production derived from iron dyshomeostasis could also affect the aggregation of further proteins observed in FTD-associated inclusions, like TAR DNA binding protein (*TARDBP/TDP43*), fused in Sarcoma (*FUS*), EWS RNA binding protein 1 (*EWSR1*), TATA-box binding protein associated factor 15 (*TAF15*), ubiquilin 2 (*UBQLN2*) and the dipeptide repeats (DPRs) derived from the translation of the hexanucleotide repeat region of the C9orf72 gene.

Mutations in further genes associated with FTD have been found to induce mitochondrial dysfunctions, although the possible effects on iron homeostasis were not investigated. Mitochondrial defects have been demonstrated in *Drosophila* models of C9orf72 ALS/FTD, in which only the reversal of oxidative stress through the overexpression of key antioxidant genes of the *KEAP1/NRF2* signaling pathway partially rescued the observed mitochondrial defects [130]. It is worth noting that *NRF2* is a transcription factor directly regulating several genes that are crucial for iron [7,12]. Mitochondrial defects were also observed in C9orf72 mutant human fibroblasts grown in galactose to induce the switch from glycolytic to oxidative metabolism, with increased oxygen consumption, ATP and reactive oxygen species (ROS) production, and mitochondria hyperpolarization [152]. Conversely, TARDBP/TDP43 mutations in these cellular model and growth conditions resulted in the decrease in mitochondrial membrane potential with no alterations in oxygen consumption rate [152]. Several further published works reported mitochondrial abnormalities in *TARDBP/TDP43* proteinopathies, showing that pathological mutations in this protein may perturb mitochondrial fission and fusion dynamics, mitochondrial trafficking, bioenergetics, and mitochondrial quality control [153]. FUS mutations resulted in the sequestration of mRNAs encoding mitochondrial respiratory chain components, which induced disorganized mitochondrial networks, reduced aerobic respiration and increased ROS [126]. A previous work further demonstrated that an ALS-associated FUS mutation disturbs the translation efficiency of mitochondrial-associated genes [154]. ALS-associated mutant FUS induced mitochondrial fragmentation in mammalian neuron-like cells, cultured neurons and transgenic flies and reduced the mitochondrial membrane potential, increasing the production of mitochondrial ROS in HEK293 cells [155]. A physiological role of FUS in mitochondrial DNA (mtDNA) repair has been recently elucidated, suggesting a novel role of FUS mutations in mitochondrial dysfunctions [156]. T-cell intracellular antigen protein 1 (TIA1) is a further gene associated with FTD, which was recently found to be involved in mitochondrial dynamics [129]. Its deficiency was related to enhanced mitochondrial activities, like mitochondrial membrane potential, ATP synthesis and cellular oxygen consumption rate and cellular senescence [157]. The ubiquilin 2 (UBQLN2) gene is also implicated in mitochondrial functions, particularly in regulating mitochondrial protein import and quality control. Transgenic mice overexpressing the P497S ALS/FTD mutation and UBQLN2 knockout cells showed reduced levels of several mitochondrial proteins involved in respiration, mitochondria complex assembly, dynamics and import, accompanied by age-dependent respiration deficits, that were related to defects in mitochondrial protein import due to the aberrant targeting of the translocase of inner mitochondrial membrane 44 (*TIMM44*) to mitochondria [158]. Several studies demonstrated mitochondrial dysfunctions also in FTD, inclusion body myopathy with early-onset Paget disease and frontotemporal dementia (IBMPFD) and ALS, which was linked to mutations in the valosin-containing protein (VCP) gene. *VCP* is a type II ATPase that is expressed both in the nucleus and cytoplasm of several tissues and is involved in several cellular functions including protein clearance, autophagy and mitophagy [159,160]. Patients’ and murine mutated primary myoblasts and fibroblasts also showed decreased respiratory capacity and decreased production of ATP [127,161,162], suggesting mitochondrial dysfunction even in this particular form of FTD. In conclusion, many experimental observations suggest the impairment of crucial mitochondrial functions and pathways in FTD and the involvement of several FTD-associated genes in the maintenance of mitochondrial homeostasis. Considering that mitochondria are iron-rich organelles that are particularly abundant in neurons and are the major site of iron handling in the cells, we may speculate that the observed dysfunctions, in addition to resulting in deficits in mitochondrial dynamics, trafficking, bioenergetics and organelle quality control, may also trigger iron homeostasis imbalance that may impact on further main mechanisms involved in FTD pathology (Figure 5).

### 5.2. Iron Homeostasis and Ferroptosis

In the latest years, ferroptosis, a novel form of programmed cell death induced by iron-dependent lipid peroxidation and prevented by iron chelation, has been observed in several neurodegenerative disorders [18]. Ferroptosis is strictly related to iron availability in the cell, since iron is an essential co-factor of lipoxygenases that catalyze the dyoxygenation of polyunsaturated fatty acids (PUFAs), and it can also directly catalyze lipid peroxidation through the Fenton reaction. The increase in cellular iron, by increased uptake from the extracellular milieau or enhanced autophagic/lysosomal degradation of ferritin by ferritinophagy, may then elicit ferroptosis [163]. While firstly studied in cancer, ferroptosis is now emerging as a trigger in cellular death in multiple diseases and several neurodegenerative disorders like AD, PD and ALS [164,165]. Although thoroughly studied in a variety of neurological conditions, very little is known, however, about the role of ferroptosis in FTD. Considering the increasing evidence of iron deposition in specific and vulnerable brain regions, it is surprising that the involvement of ferroptosis in neuronal death in FTD has not been emphasized yet. Hence, this question should be addressed in future studies. Indeed, as described above, mutations in FTD-associated genes have been found to directly or perhaps indirectly disrupt cellular iron homeostasis [54,99,102,106,145], and it is reasonable to hypothesize that they may influence or trigger the mechanisms of ferroptosis in neurons and/or glial cells.

In addition to iron availability, ferroptosis is related to multiple signaling pathways, the intracellular antioxidant system being crucial. Oxidative stress has been observed in several models of FTD [97,102,130,166,167]. The NRF2 gene is a master regulator of cellular redox homeostasis and is implicated in several cellular defense mechanisms, including the regulation of oxidative stress, inflammation and mitochondrial metabolism [168]. The *NRF2* protein is also strictly implicated in the protection against ferroptosis [169]. The transcription factor is activated by cellular oxidative stress. As stated above, under normal conditions, *NRF2* is degraded by the proteasome through *KEAP1* interaction; conversely, under oxidative stress conditions, *NRF2* is activated and translocated to the nucleus, promoting the expression of several protective genes involved in the cellular redox homeostasis and modulating iron homeostasis [7,10]. As recently described, mitochondrial oxidative stress seems to be a fundamental mechanism contributing to the pathogenesis of C9orf72 mutation in ALS/FTD, and just *NRF2* has been found to play a crucial role in rescuing the neurodegenerative phenotype in a C9orf72 *Drosophila* model and in C9orf72 patient-derived iNeurons [130]. It is then reasonable to hypothesize that ferroptosis may also be implicated in C9orf72 hexanucleotide expansion pathogenesis or related cellular death, also considering that cortical iron accumulation in glial cells has been observed in FTD patients harboring the expansion [54]. The recent emerging data on the involvement of iron homeostasis dysregulation and consequent oxidative stress in FTD, not only related to the C9orf72 mutation, may more generally suggest the engagement of ferroptotic mechanisms in this form of dementia. Antioxidant defenses are relatively low in the brain [170]. Considering the crucial role of *NRF2* in the protection against oxidative stress and ferroptosis in other neurodegenerative disorders [171,172,173,174], we may speculate that the *NRF2* signaling pathway, regulating redox balance between oxidants and antioxidants, may be crucial in protect brain cells against ferroptosis also in FTD forms associated with genetic mutations other than C9orf72 expansion. Indeed, ferroptosis may act on *MAPT/TAU* hyperphosphorylation and aggregation [175], and ferroptotic neuronal death linked to iron overload and lipid peroxidation has been hypothesized in the P301S MAPT/TAU transgenic murine model [176]. Although not yet studied for FUS-FTD-associated mutations, FUS-ALS-causing mutations lead to an increased vulnerability to ferroptosis [177] and induce mitochondrial dysfunctions accompanied by increased oxidative stress [126]. The *SQSTM1-p62*/*KEAP1*/*NRF2* signaling pathway is strongly implicated in ferroptosis inhibition, in which *SQSTM1/p62* is a protein induced by oxidative stress and activating the *NRF2* protective function [178]. Intriguingly, as above reported, the ALS- and FTD-associated G427R mutation in the SQSTM1/p62 gene has been demonstrated to disrupt the *NRF2* anti-oxidative response in cellular in vitro models [135], further suggesting the possible involvement of ferroptosis in some forms of FTD.

Ferroptosis is also characterized by mitochondrial dysfunction, although the role of mitochondria in this kind of programmed cell death is still controversial [179]. Mitochondria are an important source of ROS produced during the oxidative phosphorylation pathway and the tricarboxylic acid cycle, and several mitochondrial antioxidant enzymes play a significant role in inhibiting ferroptosis. Further, mitochondria are the main cellular site involved in iron handling, being the organelles crucially involved in the synthesis of heme and ISCs, and impaired mitochondrial iron metabolism has been shown to lead to ferroptosis. Third, mitochondrial DNA damage may trigger ferroptosis [179]. Interestingly, mitochondrial transplantation has been found to rescue neuronal cells from ferroptotic cell death [180]. As thoroughly discussed above, mitochondrial dysfunction, increased mitochondrial iron content and mitochondrial DNA damage have been implicated in FTD pathogenesis. These observations should then be considered in relation to ferroptosis in future studies on FTD pathomechanisms.

Finally, inflammation is also strictly related to ferroptosis through the activation of several inflammatory signaling pathways, including the *STING* pathway [181]. The *STING* signaling pathway is an innate immune response to double-stranded DNA released by pathogens, but it is also activated by cellular DNA damage released after cell death or mitochondrial dysfunction. As discussed above, although not yet demonstrated in neurons, cellular iron excess or dyshomeostasis may trigger *STING* activation [115,116,117]; conversely, *STING* activation may enhance *NCOA4*-driven ferritinophagy and drive harmful iron release in the cytosol [119,182]. FTD is characterized, like further neurodegenerative disorders, by neuroinflammation. Intriguingly, as mentioned above, neuronal activation of the *STING* pathway by DNA damage has recently been observed within vulnerable neurons in ALS/FTD [107], confirming previous observations in TARDBP/TDP43 models of ALS [183]. The link between neuroinflammation, the activation of the *STING* signaling pathway and cell death through ferroptosis then warrants forthcoming studies.

In conclusion, a lot of studies seem to suggest that ferroptosis may be implicated in the pathogenesis of FTD or may be a downstream effect of the pathogenetic mechanisms involved in FTD, similarly to the strictly related ALS and further neurodegenerative disorders (Figure 5). Future studies should then focus on the exploration of this cell death mechanism specifically in FTD.

### 5.3. Iron Homeostasis and Protein Aggregation

Protein misfolding and aggregation are hallmarks of several neurodegenerative diseases. *SOD1*, *TARDBP/TDP43*, *FUS*, *TAF15*, *EWSR1*, *UBQLN2*, heterogeneous nuclear ribonucleoprotein A1 (*hnRNPA1*) and heterogeneous nuclear ribonucleoprotein A2/B1 (*hnRNPA2B1*) form toxic aggregates in FTD and ALS [184,185]. The C9orf72 hexanucletide expansion mutation results in the unconventional translation of transcripts containing the hexanucleotide GGGGCC repeat; this translation produces dipeptide repeat proteins that aggregate and accumulate in the CNS of FTD and ALS patients [186]. *MAPT/TAU* forms aggregates in several tauopathies, including FTD, PSP, CBD and AD [185]. Amiloid β (*Aβ*) aggregates are also found in amyloid plaque in AD, while *SNCA* forms aggregates in synucleinopathies like PD and DLB [185]. Cellular prion protein (*PrPc*) aggregates into a detergent-insoluble and protease-resistant form, which is termed PrP-scrapie (*PrPSc*) in prion disorders [187]. Proteins involved in CAG repeat expansion disorders like HD are also prone to misfolding and aggregation [188]. It is worth noting that the aggregation of one protein is not characteristic and specific of each individual disease; as an example, *MAPT/TAU* aggregates accumulate in AD, FTD, PSP, CBS and several other neurodegenerative disorders, although different conformational strains may be responsible for each disorder [189]. Interestingly, iron has been shown to induce or enhance the aggregation process of some of the above-mentioned proteins, i.e., *MAPT/TAU*, *Aβ*, *SNCA* and *PrPc* [190]. The mechanism through which iron mediates this process is not clear, although there are two main hypotheses: a direct effect through protein binding and an indirect effect through oxidative stress.

*MAPT/TAU* inclusions in the CNS have been found in several clinically and genetically distinct neurodegenerative diseases, which are collectively named tauopathies. AD is the most common of these disorders, but *MAPT/TAU* pathology is also observed in FTD and further conditions. In primary tauopathies, the *MAPT/TAU* pathology is the main driver of neurodegeneration, like in FTLD-TAU disorders, while in secondary tauopathies, *MAPT/TAU* pathology coexists with other pathologies, like *Aβ* plaques in AD [191]. Tauopathies may be caused by different mutations in different genes. Pathogenic variants in the MAPT/TAU gene cause FTD (FTDP-17), but also PSP, corticobasal degeneration (CBD) and Pick’s disease, while these and other tauopathies have been linked to pathogenic variants in several other genes, like APP, PSEN1 and PSEN2 in AD. The *MAPT/TAU* protein is mainly involved in the stabilization of microtubules of neuronal axons and exists in six major isoforms, which are derived from alternative splicing and about equally expressed in the healthy CNS. Clinically distinct tauopathies can be classified also based on the isoforms that accumulate in the inclusions; further, some MAPT/TAU pathogenic mutations can modify the appropriate ratio of these isoforms, leading to an altered ability of the protein to stabilize microtubules. The *MAPT/TAU* protein is also normally subjected to post-translational modifications, like phosphorylation. Phosphorylation is involved in regulating the physiological functions of *MAPT/TAU*, including its binding to microtubules and their stabilization and assembly. Although *MAPT/TAU* is a positively charged and highly soluble protein, increased or altered phosphorylation, improper further post-translational modifications (like glycation and nitration), interaction with polyanions and some pathological mutations (like P301L) are involved in its impaired interaction with microtubules, its reduced solubility of the natively unfolded and intrinsically disordered protein, its fibrillization, and its aggregation [192]. Iron, whose accumulation is observed in some tauopathies like AD and PSP, has been demonstrated to bind *MAPT/TAU* and promote or enhance its aggregation. Iron was first demonstrated to bind *MAPT/TAU* in a redox-active manner [193]. Nonetheless, it is not currently clearly defined if Fe^3+^, Fe^2+^ or both ions may trigger *MAPT/TAU* aggregation. Yamamoto et al. [194] demonstrated that this aggregation is induced in vitro only by Fe^3+^, while it is reversed by reducing Fe^3+^ to Fe^2+^. Iron chelators like deferoxamine (DFO) and Feralex-G were demonstrated to reverse this interaction [195]. The interaction of Fe^3+^ but not divalent ions with *MAPT/TAU* and its ability to cause conformational changes and induce protein aggregation in vitro was further confirmed in successive studies [196,197,198]. However, by electrochemical studies, Ahmadi et al. [199] demonstrated that both Fe^2+^ and Fe^3+^ can bind the *MAPT/TAU* protein and induce conformational changes in the protein that lead to aggregation and that are more pronounced with Fe^2+^ interaction. A recent ex vivo study demonstrated elevated iron in the inferior temporal cortex of AD subjects with a strong association with the rate of cognitive decline but a weak association with the extent of *MAPT/TAU* neurofibrillary tangles, suggesting the hypothesis that iron, accumulating with the aggregates, may potentiate neurodegeneration [200]. A subsequent in vivo study demonstrated a close relationship between iron accumulation and pathological *MAPT/TAU* aggregation by imaging techniques in AD patients, further suggesting a modulatory effect of iron burden on the disease process [201]. Finally, Chen et al. [202] showed that a high dietary iron content resulted in iron accumulation in the hippocampal dentate gyrus region of APP/PSEN1 double transgenic mice, in turn resulting in *MAPT/TAU* accumulation, that may impact on protein aggregation. Iron has also been shown to modulate *MAPT/TAU* phosphorylation and consequently its aggregation. Within the amino acid sequence of *MAPT/TAU*, >85 putative phosphorylation sites are predicted, but not all are found to be effectively modified. Although the addition of a phosphate group, altering the charge of the protein, may also alter its conformation, it should also be taken into account that the phosphorylated sites prevalently found in aggregated *MAPT/TAU* in neurodegenerative disorders are also modified, although to a different extent, in functional soluble *MAPT/TAU*. Then, the causal relationship between phosphorylation and *MAPT/TAU* aggregation is still a matter of debate [203]. The overexpression of the heme oxygenase 1 (HMOX-1) gene may induce the phosphorylation and aggregation of *Mapt/Tau* by releasing free iron and enhancing iron loading in mouse brain and murine Neuro2a cells [204]. Several kinases have been implicated in pathogenic *MAPT/TAU* phosphorylation, which include *GSK3β*, activated by *Aβ*, and the cyclin-dependent kinase-5 (*CDK5*), whose activity is regulated by its binding with neuron-specific proteins. Among these regulatory proteins, the p25 proteolytic fragment of p35 is increased in AD brains, leading to the hyperactivation of *CDK5* and *MAPT/TAU* hyperphosphorylation and aggregation. Egaña et al. [205] demonstrated that in rat hippocampal neurons, iron induced cell damage by oxidative stress through lipid peroxidation, and this effect was correlated with the depletion of p25, hypoactivation of *CDK5* and the decrease in *MAPT/TAU* phosphorylation. Lovell et al. [206] showed that iron-induced oxidative stress increased *MAPT/TAU* phosphorylation in primary rat cortical neuron cultures through *GSK3β* hyperactivation. Several further studies suggested an effect of iron accumulation on the hyperactivation of those kinases in several models, in turn resulting in *MAPT/TAU* phosphorylation and aggregation, and some of these studies also suggested the use of metal chelators to attenuate *MAPT/TAU* accumulation [141,207]. Based on these findings, it may also be speculated that the role of iron in the enhancement of *MAPT/TAU* phosphorylation may be through the derived oxidative stress [208]. Further, iron excess may also promote *MAPT/TAU* hyperphosphorylation through other signaling pathways like the insulin one [209]. In conclusion, iron binds *MAPT/TAU*, causes conformational changes in the protein, and promotes its hyperphosphorylation and aggregation, leading to neurotoxicity and neuronal death. Although most, if not all the above-described studies are relative to AD pathology, we can speculate similar effects also in FTD caused by MAPT/TAU mutations and, more broadly, with *MAPT/TAU* pathology.

As stated above, iron has also been shown to enhance the aggregation process of *Aβ*, *SNCA* and perhaps *PrPc*, in AD, PD and spongiform encephalopathies, respectively [190]. Since these proteins are not strictly involved in FTD pathology, we only briefly review the main evidence of these interactions, referring the readers to more comprehensive reviews on this topic [190,210,211,212].

Apart from intra-neuronal neurofibrillary tangles formed by hyperphosphorylated *MAPT/TAU*, AD is characterized by the accumulation of *Aβ* aggregates in extracellular plaques, which are derived from the amyloidogenic processing of *APP*. *APP* is directly involved in cellular iron homeostasis by stabilizing *FPN1/SLC40A1* and acting as a ferroxidase in the brain, then promoting iron efflux from neurons; as described above, *MAPT/TAU* is implicated in this role of *APP* [139]. In turn, iron regulates *APP* expression through the IRE/IRP regulation system [213] and *APP* cleavage through the amyloidogenic and non-amyloidogenic processing pathways [214]. Then, iron loading can both upregulate *APP* expression and induce its amyloidogenic processing, directly contributing to *Aβ* pathology in AD. Indeed, iron levels are elevated in AD patients’ brain in association with not only *MAPT/TAU* pathology but also *Aβ* plaques [190,215]. *Aβ*, apart from being involved in AD pathology, has also physiological functions, regulating neurogenesis, synaptic plasticity, memory formation, and calcium homeostasis, and it has also antioxidant properties through its role in metal sequestration and the prevention of ROS production [216]. On the other hand, *Aβ* has also been demonstrated not only to bind and sequester iron but also to reduce Fe^3+^ to Fe^2+^, in turn generating oxidative stress [217]. Iron has also been found to promote *Aβ* aggregation, contributing to AD pathology, while iron chelation has been found to reduce *Aβ* aggregation, similarly to what happens in *MAPT/TAU* pathology. Probably the work by Dyrks et al. [218] was the first evidence of the involvement of iron in *Aβ* aggregation. Since then, several further studies have approached this issue and demonstrated the role of iron in promoting *Aβ* aggregation [190,215]; one of the more recent works demonstrated that chemically reduced pure ferrous iron is directly associated with amyloid pathology, and diffuse amyloid deposits comprise an iron–amyloid composite [219].

*SNCA* is a small protein highly expressed in the brain and involved in synaptic vesicle trafficking and homeostasis. *SNCA* oligomers and larger aggregates are a pathological hallmark of PD and further α-synucleinopathies. *SNCA* is an intrinsically disordered protein that forms α-helical structures upon binding to lipids through its amphipathic N-terminal region. The polar C-terminal region includes post-translation modification sites and mediates the interaction of *SNCA* with other proteins. The central hydrophobic region can adopt β-sheet conformation and form amyloid-like fibrils, that localize in Lewy bodies and Lewy neurites, which are found in α-synucleinopathies. First, *SNCA* forms transient soluble oligomeric protofibrils; then, protofibrils become insoluble and aggregate in an insoluble form. It is generally assumed that oligomeric protofibrils are neurotoxic, while mature fibrils seem to play a role in the spread and progression of the disease. Like *MAPT/TAU*, *SNCA* is subject to multiple post-translational modification. Among these modifications, phosphorylation and oxidation may modulate aggregation. Several missense mutations in the SNCA gene and its duplications and triplications are among the known pathogenic mutations in PD and affect the process of aggregation [220]. Like *APP*, *SNCA* is directly involved in iron homeostasis, being a cellular ferrireductase that, in the presence of NADH and binding copper, can reduce Fe^3+^ to Fe^2+^, harmfully increasing cellular Fe^2+^ levels. SNCA mutations, however, only slightly affect the enzymatic activity [221]. Further, again like *APP*, *SNCA* expression is post-translationally regulated by iron, containing in the 5′-UTR of its mRNA an IRE-like sequence, whose regulation can enhance *SNCA* translation in iron overload conditions, probably contributing to its pathological aggregation [222]. Several studies demonstrated iron dysregulation and accumulation in PD and α-synucleinopathies, particularly in dopaminergic neurons of substantia nigra, and they are excellently summarized in several reviews to which we refer the readers for a more detailed description [211,223]. *SNCA* has also been found to bind iron, like *MAPT/TAU* and *APP*. This interaction may also contribute to *SNCA* aggregation. The interaction of the C-terminal region of *SNCA* with Fe^2+^ was demonstrated by NMR spectroscopy and was accompanied by enhanced protein aggregation in vitro [224]. *SNCA* interaction with Fe^2+^ but also with Fe^3+^ was further demonstrated in several studies [221,225]. Importantly, several studies highlight the role of iron and iron-induced oxidative stress in *SNCA* aggregation. In BE-M17 neuroblastoma cells overexpressing the wild-type *SNCA*, iron exposure induced the formation of intracellular aggregates that contain *SNCA* and ubiquitin. The overexpression of mutant *SNCA* (A53T and A30P) resulted in more pronounced aggregation after treatment with FeCl_2_ [226]. In vitro, Fe^3+^ induced a conformational change in *SNCA* and accelerated the rate of fibril formation [227]. Golts et al. [228] and Barathi et al. [229] observed that different metals modulated in different ways the conformation, fibril morphology and aggregation of *SNCA*. Kostka et al. [87] further demonstrated protein aggregation in vitro under conditions of low micromolar concentrations of Fe^3+^ and low nanomolar concentrations of *SNCA*. The ability of Fe^3+^ to promote *SNCA* fibrillation at low concentration was confirmed by Zhao et al. [230], who further demonstrated that high Fe^3+^ concentrations can conversely inhibit the conversion to fibrils. The researchers also demonstrated that H50 and E57 residues are involved in Fe^3+^ binding to the protein and that H50 mutation abolishes *SNCA* fibrillation induced by Fe^3+^. The effect of iron (both Fe^2+^ and Fe^3+^) on *SNCA* aggregation was demonstrated to be only partially linked to oxidative stress in SK-N-SH cells [231], while Levin et al. [232] observed in vitro that oligomer formation is due to a direct interaction of Fe^3+^ with *SNCA*, but ROS enhance aggregation through the oxidation of Fe^2+^ to Fe^3+^. In *Drosophila melanogaster* exposed to iron treatment, Zhu et al. [233] showed that the overexpression of mutant (A53T, A30P) *SNCA* results in a more severe motor deficit and selective dopaminergic neuron loss in comparison to the overexpression of the wild-type protein. Conversely, Agostini et al. [234] demonstrated that the overexpression of wild-type *SNCA* within dopaminergic neurons of *Drosophila melanogaster* induces protein aggregation, the degeneration of dopaminergic neurons, locomotor deficit and reduction in lifespan that were exacerbated by iron overload and attenuated by iron chelators and antioxidant treatment with N-acetylcysteine, also suggesting the involvement of ferroptosis in *SNCA* toxicity. Furtherly, under aerobic conditions, Fe^2+^ was found to promote the right-twisted antiparallel β-sheet oligomerization of N-terminally acetylated *SNCA*, the physiologically relevant form of the protein, while under anaerobic conditions, iron induced parallel β-sheet oligomerization [235]. Iron was also shown to promote *SNCA* aggregation by inhibiting the transcription factor EB (*TFEB*), which is the master regulator of the autophagosome-lysosome pathway [236].

Prion disorders, like Creutzfeldt–Jakob disease (CJD), are a peculiar group of sporadic, familial or transmissible neurodegenerative disorders characterized by the accumulation in the brain of aggregates of *PrPc* in a detergent-insoluble and protease-resistant form, which is termed *PrPSc*. *PrPc* is codified by the prion protein (PRNP) gene, and it is abundantly expressed in the brain, neurons and glial cells. *PrPc* is a membrane glycosylphosphatidylinositol-anchored glycoprotein mainly involved in the maintenance of peripheral myelin, neurite outgrowth and neuronal signaling. Its aggregation is due to a conformational change in *PrPc* in a β-sheet-rich form, named *PrPSc*, due to spontaneous events in sporadic forms, mutations of the PRNP gene in familial forms or a direct contact with exogenous *PrPSc* in the transmissible forms of these neurodegenerative disorders. Further proteins aggregate together with *PrPSc*, including ferritin, which is found in CJD diseased brains, resulting in the sequestration of iron in a biologically unavailable form, increase in total iron and, paradoxically, an iron-deficient phenotype [187,212,237,238]. Brain iron homeostasis is then disrupted in prion disorders, as described in several published works thoroughly reviewed elsewhere [187,239]. *PrPc* itself is also directly involved in iron homeostasis, functioning as a plasma membrane ferrireductase and inducing the uptake of *TF* and non-*TF*-bound iron [240,241,242]. Similarly to APP and SNCA genes, PRNP expression is post-translationally regulated by iron through an IRE-like sequence within the 5’-UTR of its mRNA [85]. Through its octapeptide region, *PrPc* interacts with divalent cations like copper, zinc, nickel and manganese; however, the evidence for a direct binding of the protein domain with further metals like iron remains elusive [243]. Regarding *PrPc* aggregation, a few studies focused on iron involvement. Using the protein misfolding cyclic amplification (PMCA) technique, FeCl_2_ was found to induce the conversion of *PrPc* into a protease-resistant aggregated form [244]. Basu et al. [245] demonstrated that *PrPc* binds iron both in vitro and in vivo in M17 human neuroblastoma cells overexpressing *PrPc*, likely forming a *PrPSc*–ferritin complex. The authors also showed that the exposure of cells to FeCl_2_ causes the upregulation of *PrPc*, its conversion to a *PrPSc*-like form and the aggregation of additional *PrPc*, simulating *PrPSc*. The involvement of iron in *PrPc* aggregation was further demonstrated after the exposure of human neuroblastoma cells overexpressing wild-type and mutant *PrPc* to ferric ammonium citrate; this aggregation was explained as a cytoprotective response to redox-active iron, although the subsequent co-aggregation of *PrPc* with ferritin was found to induce cellular toxicity [246]. Finally, Choi et al. [247] showed that HpL3-4 murine hippocampal cells treated with exogenous recombinant *PrPc* internalized more recombinant protein when exposed to ferric ammonium citrate than to FeCl_2_. The researchers also showed that *PrPc* accumulates in the insoluble cellular fraction in a protease-resistant form during endosomal vesicular trafficking and not through a direct contact between *PrPc* and Fe^3+^.

Regarding further aggregated proteins involved in neurodegenerative disorders, and particularly in FTD, to the best of our knowledge, no evidence supports the direct role of iron in the process of aggregation. Nonetheless, iron could trigger protein aggregation indirectly. SOD1 mutations have an impact on iron homeostasis, while iron chelators demonstrated therapeutic effects and reduced *TARDBP/TDP43* aggregation in SOD1 murine models of ALS [248]. Iron can catalyze ROS formation through the Fenton reaction; then, an indirect effect of iron on protein aggregation may act through oxidative stress, which was thoroughly described in several neurodegenerative disorders like FTD. Cysteine residues within proteins are particularly susceptible to oxidative stress-induced post-translational modifications, which may influence protein misfolding and aggregation [249]. Oxidative stress was indeed found to promote *TARDBP/TDP43* aggregation through cysteine oxidation and disulfide bond formation between conserved cysteine residues within the N-terminal region and within and surrounding the second RNA-recognition motif of the protein [250,251]. The oxidative modification of cysteine was also found to promote *SOD1* disulfide bond-dependent and -independent aggregation [252,253]. Interestingly, disulfide bridges are also involved in *MAPT/TAU* aggregation [254,255]. Glutathionylation is a redox-sensitive post-translational modification that can alter the stability of the target proteins. Oxidative stress is known to promote the localization of *FUS* to cytoplasmic stress granules and promote its aggregation. Interestingly, the glutathionylation of *FUS* cysteine 447 was found to induce *FUS* aggregation in *Drosophila* brains [256]. Further, heme metabolism dysfunctions have been linked to several neurodegenerative disorders; heme itself and iron released during heme degradation by *HMOX1* may trigger oxidative stress [257]. Intriguing recent research furtherly showed that heme may impair protein degradation by proteasome, promoting the formation of perinuclear aggresomes of ubiquitinated proteins and leading to unfolded protein response in mouse embryo fibroblasts (MEF) isolated from mice, both wild-type and lacking Hmox1. Proteasome impairment may be particularly deleterious in neurodegenerative disorders like FTD in which the clearance of unfolded and aggregated proteins is crucial [258]. Furtherly, heme and iron were demonstrated to induce the formation of *SQSTM1/p62*^+^ and ubiquitin^+^ protein aggregates known as aggresome-like induced structures (ALIS) in macrophages, which were formed as part of the cellular response to oxidative stress driven by the *NRF2* transcription factor [259].

In conclusion, strong experimental evidence demonstrated the direct or indirect role of iron in promoting *MAPT/TAU* aggregation; although most studies were conducted considering AD pathology, we can speculate similar mechanisms in FTD pathology. Oxidative stress, that may be related to dysfunctions of iron homeostasis, could affect the aggregation of some FTD-associated proteins like *TARDBP/TDP43* and *FUS,* suggesting the indirect involvement of iron in triggering aggregation, although this potential mechanism has not yet been investigated. Further proteins involved in FTD pathology may aggregate and cause neurotoxicity. The dipeptide repeats observed with the C9orf72 hexanucleotide expansion have been found to form toxic oligomers and amyloid-like aggregates in FTD patients [260,261,262], and mutant *UBQLN2*, *TAF15* and *EWSR1* may also self-aggregate [263,264]. The huge amount of the above-reviewed research work may suggest future investigations also in the case of unexplored protein aggregates involved in FTD pathology (Figure 5).

## 6. Conclusions

The disruption of iron homeostasis has been thoroughly demonstrated in several neurodegenerative disorders. Nonetheless, its contribution in the pathomechanisms of FTD are still largely unexplored, although recent studies have begun to investigate this hypothesis. FTD-associated genes may play a critical direct or indirect role in brain iron handling, regulating mitochondrial functions, modulating neuroinflammation and oxidative stress or controlling ER–mitochondria communications and the lysosome–autophagy pathways. Iron dyshomeostasis could contribute to neurodegeneration through the induction of mitochondrial dysfunctions, protein misfolding and aggregation or through the activation of ferroptosis. Not least, considering the recently described accumulation of iron in the brain of FTD patients, iron chelation might offer a new therapeutic approach in this neurodegenerative disorder. Deferiprone (DFP) seems to be the most promising iron-chelating agent for the brain due to its iron-relocating and redistributing abilities and because it can cross the BBB. Chelating agents have been clinically evaluated to target brain iron loading in several brain disorders, like FRDA, NBIA, PD, ALS and AD, in some cases with neuroprotective results; however, in others, they have shown detrimental effects [164,190,265,266,267,268,269]. Further research in these directions is critical to provide a deeper understanding of the currently underestimated role of this essential but harmful transition metal.

## Figures and Tables

**Table 1:** Summary of the current findings related to evidence of iron involvement in FTD based on clinical, imaging, histological and biochemical studies.

Findings (Methods)	References
increased iron content in post-mortem brains (INAA ^1^)	[42]
severe microglial activity in frontal and temporal cortex (ferritin immunohistochemistry)	[43]
aberrantly regulated ferritin in frontal cortex (2D gel electrophoresis and MALDI-TOF ^2^)	[44]
overlapping with NBIA (clinical findings)	[45,46,47,48]
increased iron load in claustrum, caudate nucleus, putamen, globus pallidus, thalamus and subthalamic nucleus in FTLD-FUS and FTLD-TDP (MRI ^3^)	[49]
increased iron deposition in basal ganglia and cortical micro-bleeds in FTLD-FUS and FTLD-TDP (MRI ^3^ and histopathological examination)	[50]
iron deposition and atrophy in basal ganglia of patients harboring the H63D mutation in the HFE ^4^ gene (MRI ^3^)	[51]
increased iron levels in bilateral superior frontal and temporal gyri, anterior cingulate, putamen, right precentral, right insula, right hippocampus and right red nucleus; positive association between apathy and iron content in the superior frontal gyrus and between disinhibition and iron content in the putamen in patients with bv-FTD (SWI ^5^)	[52]
iron-rich superficial cortical layer astrocytic processes surrounding small blood vessels; dystrophic patterns of punctate iron-rich microglia in gray matter in FTLD-TDP sporadic cases; iron-positive ameboid and hypertrophic microglia and astrocytes in deeper gray matter and adjacent white matter in FTLD-tau sporadic cases (ex vivo MRI ^3^ and histopathological study)	[53]
cortical iron accumulation in activated, dystrophic microglia and reactive astrocytes, associated with the severity of proteinopathy and neurodegeneration in FTD-tau cases due to MAPT/TAU mutations and FTD-TDP cases due to large expansion mutation in the C9orf72 gene (MRI ^3^ and histopathological study)	[54]
severe *TARDBP/TDP43* pathology and focal iron accumulation in the precentral gyrus and frontal operculum in an ALS patient presenting with speech apraxia as early symptom (SPECT ^6^, MRI ^3^, histopathological study)	[55]
decreased *CP* ^7^ enzymatic activity/*CP* ^7^ content ratio in PPA patients (serum biochemical analyses)	[57]

^1^ instrumental neutron activation analysis. ^2^ matrix-assisted laser desorption/ionization time-of-flight mass spectrometry. ^3^ magnetic resonance imaging. ^4^ homeostatic iron regulator. ^5^ susceptibility-weighted imaging. ^6^ single-photon emission computed tomography. ^7^ ceruloplasmin.

**Table 2:** FTD-causative genes and main findings related to iron homeostasis.

Gene	Protein Localization	Experimental Condition	Findings	References
CHCHD10	mitochondrion	in vitro by gene silencing in HEK293 cells	increased mitochondrial iron content	[99]
CHMP2B	ESCRT-III ^1^	in vitro in human iPSCs from patients carrying the 31449G>C splicing mutation (forebrain cortical neurons)	misexpression of iron-related genes, increased cytoplasmic Fe^2+^ levels	[102]
C9orf72	cytoplasmic vesicles and organelles, nucleus	bioinformatic analysis	5′-UTR IRE-like sequence	[105]
		in vitro	complexes of RNA and DNA G-quadruplexes with heme with enhanced peroxidase and oxidase activity	[106]
		patients	cortical iron accumulation in activated and dystrophic microglia and reactive astrocytes	[54]
MAPT/TAU	cytoskeleton	patients	cortical iron accumulation in activated and dystrophic microglia and reactive astrocytes	[54]
		knockout mice in vitro (knockout murine primary neuronal cultures)	age-dependent brain atrophy and neurodegeneration with iron accumulation in the cortex, hippocampus and substantia nigra, iron accumulation through the impairment of APP ^2^ trafficking and APP ^2^-FPN/SLC40A1 ^3^ interaction	[139,140]
		in vivo by the overexpression of human full-length MAPT/TAU in hippocampal CA3 region of mice and in vitro in neuronal cell cultures (SHSY-5Y and N2a cell lines)	iron accumulation	[141]
SQSTM1/p62	cytoplasmic vesicles	in vitro, in vitro in cell lines (HEK293, HeLa cell lines, mouse embryonic fibroblasts, *p62*−/− mouse primary hepatocytes) (FTD-associated mutations) and in vivo (ATG7 ^4^-deficient mice)	presence of ARE ^5^ sequence in its promoter (like several iron-related genes) inactivation of KEAP1 ^6^ and activation of NRF2 ^7^ and the ARE ^5^-dependent target genes	[132,133,134,135,136]
OPTN	cytoplasmic vesicles	in vitro (knockdown HeLa cell line)	disruption of *TF* ^8^ uptake and *TF* ^8^*/TFRC* ^9^ endosomal recycling	[144]
TBK1	cytoplasm	in vitro in cell lines (FIP200 ^10^-TBK ^11^ double knockout H4 neuroglioma cells) (FTD-associated mutation)	abrogation of *TBK* ^11^ ability to drive iron release from ferritin	[145]
TMEM106B	late endosome and lysosome membranes	silencing in vitro and in vivo (MPTP ^12^/MPP^+ 13^ treated SH-SY5Y cell line and mice)	attenuation of iron accumulation in cells and in the substantia nigra of the murine model	[147]

^1^ endosomal sorting complexes required for transport III. ^2^ amyloid beta precursor protein. ^3^ ferroportin1/solute carrier family 40 member 1. ^4^ autophagy related 7. ^5^ antioxidant responsive element. ^6^ kelch-like ECH associated protein 1. ^7^ NFE2 like bZIP transcription factor 2. ^8^ transferrin. ^9^ transferrin receptor. ^10^ RB1 inducible coiled-coil 1. ^11^ TANK binding kinase 1. ^12^ 1-methyl-4-phenyl-1,2,3,6-tetrahydropyridine. ^13^ 1-methyl-4-phenylpyridinium.

## Data Availability

Due to the nature of this work, no new data were generated. The data can be obtained from the respective original papers.
